# Effect of Microcurrent Stimulation on Cartilage Regeneration in Engineered Chondrocyte Spheroids

**DOI:** 10.34133/research.1462

**Published:** 2026-10-19

**Authors:** Yong Xu, Siqiang Zheng, Qiangsheng Hu, Bo Tao

**Affiliations:** Department of Thoracic Surgery, Shanghai Pulmonary Hospital, School of Medicine, Tongji University, Shanghai, China.

## Abstract

Microcurrent stimulation has emerged as a promising biophysical cue for cartilage regeneration, but its dose-dependent effects in 3-dimensional engineered cartilage constructs remain unclear. Here, we investigated the effects of continuous direct microcurrent stimulation on auricular chondrocytes and engineered cartilage spheroids using 0-, 10-, 25-, 50-, 100-, and 200-μA stimulation for 30 min daily. In 2-dimensional culture, microcurrent stimulation did not markedly increase the DNA content or Cell Counting Kit-8 value, indicating that it did not markedly promote cell expansion or overall metabolic activity. 5-Ethynyl-2′-deoxyuridine and Ki67 assays showed that low to moderate (<50 μA) stimulation had limited effects on proliferation, whereas 100 to 200 μA attenuated proliferative activity. Nevertheless, microcurrent stimulation enhanced chondrogenic marker expression in a biphasic manner, with type II collagen, SRY-box transcription factor 9, aggrecan, and cartilage oligomeric matrix protein increasing from 0 to 50 μA and declining from 50 to 200 μA. In 3-dimensional spheroids, 50 μA produced the most favorable morphology, cartilage-specific extracellular matrix deposition, mechanical properties, and hyaline cartilage-like phenotype. Excessive stimulation increased catabolic, inflammatory, and apoptosis-associated molecular responses without causing obvious cytotoxicity, apoptosis, reactive oxygen species production, temperature change, or pH alteration. Mechanistically, 50 μA induced coordinated Ca^2+^ signaling and promoted nuclear factor of activated T cells (NFAT) nuclear translocation, whereas 200 μA generated dysregulated Ca^2+^ responses and inefficient NFAT activation. Verapamil, 1,2-bis(2-aminophenoxy)ethane-*N*,*N*,*N*′,*N*′-tetraacetic acid acetoxymethyl ester, and cyclosporin A attenuated 50-μA-induced NFAT activation, supporting the involvement of the Ca^2+^/calcineurin/NFAT pathway. Furthermore, 50-μA-preconditioned spheroids showed superior cartilage-forming capacity after subcutaneous implantation. These findings identify 50 μA as a favorable bioelectrical stimulation condition within the tested intensity range and provide a practical strategy for enhancing cartilage spheroid maturation in cartilage tissue engineering.

## Introduction

Cartilage defects remain a major clinical challenge because adult cartilage has very limited intrinsic healing capacity once its structural integrity is disrupted [1,2]. In cartilage tissue engineering, successful regeneration depends not only on the survival of implanted chondrocytes but also on the stable maintenance of the chondrogenic phenotype and sustained deposition of cartilage-specific extracellular matrix (ECM), particularly type II collagen (COL II) and glycosaminoglycans (GAGs) [3,4]. Although chondrocyte-based strategies remain among the most biologically relevant approaches for cartilage repair, their regenerative performance is still constrained by culture-induced dedifferentiation, phenotypic instability, and insufficient matrix production after expansion or implantation [5,6].

To address these limitations, 3-dimensional (3D) spheroid or microtissue systems have attracted increasing attention in cartilage regeneration [7,8]. Compared with conventional 2-dimensional (2D) monolayer culture, spheroid-based systems better preserve cell–cell interactions, more closely recapitulate the native chondrocyte microenvironment, and provide a more favorable context for matrix retention and tissue maturation [9,10]. In addition, spheroid-based cartilage constructs are particularly attractive for minimally invasive implantation and injectable regenerative strategies [11]. However, despite these advantages, engineered cartilage spheroids still face important bottlenecks, including inadequate ECM accumulation, incomplete maturation, and vulnerability to catabolic, inflammatory, and stress-related cues during long-term culture and postimplantation remodeling [12,13].

Given the persistent challenges of phenotypic instability and insufficient functional maturation in cartilage regeneration, biophysical stimulation has emerged as a promising strategy for overcoming these barriers [14]. Compared with biochemical stimulation approaches that rely on exogenous factors, electrical stimulation provides a tunable physical cue and is especially attractive because cartilage is an electroresponsive tissue and because bioelectric cues can regulate cell metabolism, matrix synthesis, and tissue remodeling [15]. In particular, microcurrent stimulation offers several practical advantages, including mild intensity, precise parameter control, and reduced risk of excessive electrical stress compared with conventional direct current or pulsed electrical stimulation approaches. Unlike piezoelectric stimulation, which generates electrical cues through mechanical-to-electrical conversion, microcurrent stimulation provides direct and controllable electrical regulation for engineered tissue maturation [16,17]. Nevertheless, the current literature still has several important deficiencies [18]. First, many published studies focus mainly on short-term cellular responses or inflammatory modulation, whereas systematic evaluation of cartilage-specific matrix formation remains limited [19]. Second, most available data are still derived from 2D culture systems, which do not adequately represent the 3D microenvironment or signal distribution experienced by engineered cartilage constructs [20]. Third, the biologically relevant dose window of microcurrent stimulation remains poorly defined, and the transition from beneficial stimulation to detrimental overstimulation is still unclear [21]. Therefore, systematic screening of stimulation intensities is necessary to identify a suitable bioelectrical stimulation range that promotes cartilage-specific matrix formation while minimizing potential adverse cellular responses. In this study, a current intensity range of 0 to 200 μA was selected to encompass low-, moderate-, and relatively high-intensity stimulation conditions, allowing evaluation of the dose-dependent biological effects of microcurrent stimulation.

This unresolved dose dependence is not merely a technical issue, but a central biological question. In cartilage engineering, an ideal stimulation strategy should enhance matrix synthesis without indiscriminately increasing cell proliferation, because excessive proliferation is often associated with dedifferentiation and loss of chondrogenic phenotype [22]. Therefore, it is essential to distinguish whether microcurrent primarily promotes cell expansion or instead improves the anabolic and functional state of chondrocytes. At the same time, increasing evidence suggests that ion channels and Ca^2+^ homeostasis play important roles in cartilage biology, matrix integrity, inflammatory responses, and degeneration, making Ca^2+^-dependent signaling a plausible mechanism by which electrical cues regulate chondrocyte behavior [23]. However, whether microcurrent-regulated cartilage matrix formation in engineered chondrocyte spheroids is mediated by a Ca^2+^-dependent signaling pathway, and whether excessive stimulation instead induces Ca^2+^ overload, oxidative stress, catabolic activation, and apoptosis-related impairment, has not been systematically clarified.

In this study, we addressed these knowledge gaps by using engineered chondrocyte spheroids as a 3D cartilage regeneration model and systematically investigating the dose-dependent effects of continuous direct microcurrent stimulation. By integrating 2D cell-level assessment, 3D spheroid maturation analysis, mechanistic interrogation, and in vivo subcutaneous implantation, we aimed to determine how microcurrent influences chondrocyte proliferation, functional activity, anabolic matrix synthesis, catabolic and apoptotic responses, and tissue-level cartilage regeneration. We further sought to test the hypothesis that optimal microcurrent stimulation enhances cartilage matrix formation through Ca^2+^/calcineurin/nuclear factor of activated T cells (NFAT) signaling, whereas excessive stimulation leads to dysfunctional Ca^2+^ signaling and impaired chondrogenesis.

The present study is innovative in several respects. First, rather than treating electrical stimulation as a generally beneficial cue, this work defines a biologically meaningful dose-dependent framework for microcurrent stimulation in engineered cartilage spheroids and clarifies the boundary between anabolic enhancement and stress-related damage. Second, unlike many previous studies centered on 2D culture, this study compared microcurrent responses in both 2D monolayers and 3D spheroid-based cartilage models, revealing stronger matrix-related regulatory effects in the 3D system. Third, this work links microcurrent-regulated cartilage formation to a specific mechanistic axis centered on Ca^2+^/calcineurin/NFAT signaling while also integrating oxidative stress, catabolic activation, and apoptosis as key determinants of overstimulation-induced failure. Finally, by combining in vitro mechanistic evidence with in vivo subcutaneous evaluation, this study extends the interpretation of microcurrent stimulation from cellular responses to cartilage-forming potential while recognizing that whether the beneficial effects of microcurrent stimulation persist after stimulation withdrawal requires further investigation and that the ectopic subcutaneous model does not fully recapitulate the mechanical environment of articular cartilage. Therefore, the significance of this work lies not only in identifying an optimal electrical stimulation window for engineered cartilage maturation but also in providing mechanistic insights into spheroid-based cartilage formation and maturation and future tissue engineering applications.

## Results and Discussion

### Microcurrent stimulation did not promote chondrocyte proliferation in 2D culture, whereas high-intensity stimulation attenuated proliferative activity

To preliminarily investigate the biological effects of microcurrent stimulation on chondrocytes, a 2D monolayer culture model was established and exposed to different current intensities (Fig. 1A). At day 9, total DNA content remained comparable among the 0-μA control group and the 10-, 25-, 50-, 100-, and 200-μA groups, indicating that microcurrent stimulation did not markedly increase the total cell number (Fig. 1B). Consistently, longitudinal Cell Counting Kit-8 (CCK-8) analysis showed comparable optical density values among all groups from day 0 to day 11, suggesting that microcurrent stimulation did not induce a sustained increase in cellular metabolic activity (Fig. S1). In agreement with this observation, CCK-8 analysis at day 5 showed no significant differences among groups, further indicating that microcurrent stimulation did not markedly alter the overall metabolic activity of chondrocytes under the present 2D culture conditions (Fig. 1C).

**Fig. 1.**
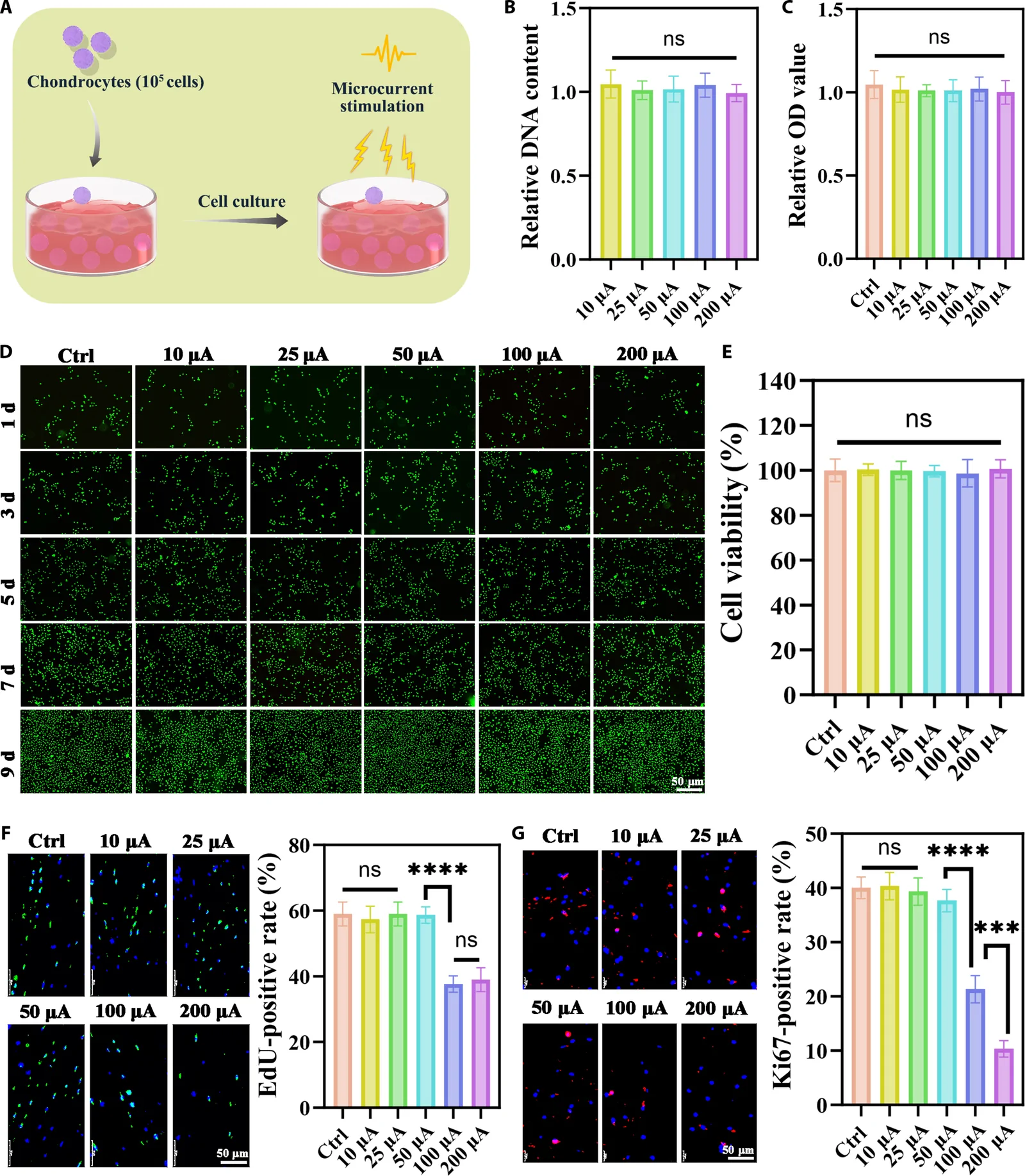
Effects of microcurrent stimulation on chondrocyte proliferation in a 2-dimensional (2D) in vitro culture system. (A) Schematic illustration of chondrocytes cultured in 6-well plates under microcurrent stimulation. (B) Relative DNA content of chondrocytes in the 10-, 25-, 50-, 100-, and 200-μA groups compared with that of the control (Ctrl, 0 μA) group at day 9. (C) Relative optical density (OD) values of chondrocytes in the Ctrl and 10-, 25-, 50-, 100-, and 200-μA groups at day 5, as determined by the CCK-8 assay. (D) Representative Live/Dead staining images of chondrocytes in the Ctrl and 10-, 25-, 50-, 100-, and 200-μA groups from days 1 to 9. (E) Quantitative analysis of cell viability in the Ctrl and 10-, 25-, 50-, 100-, and 200-μA groups at day 9. (F) Representative 5-ethynyl-2′-deoxyuridine (EdU) staining images and corresponding quantification of the EdU-positive rate of chondrocytes in the Ctrl and 10-, 25-, 50-, 100-, and 200-μA groups at day 9. (G) Representative Ki67 staining images and corresponding quantification of the Ki67-positive rate of chondrocytes in the Ctrl and 10-, 25-, 50-, 100-, and 200-μA groups at day 9. Data are presented as mean ± SD (*n* = 5). ns, not significant; ***P < 0.001; ****P < 0.0001.

Live/Dead staining showed abundant viable cells and few dead cells in all groups from day 1 to day 9, with an overall increase in viable cell coverage over time (Fig. 1D). Quantitative analysis of Live/Dead staining at day 9 further confirmed that the selected range of microcurrent stimulation did not induce obvious cytotoxicity (Fig. 1E), supporting the general biocompatibility of the stimulation protocol.

However, proliferation-specific assays revealed a more intensity-dependent response. 5-Ethynyl-2′-deoxyuridine (EdU) staining showed that low to moderate microcurrent stimulation had little effect on DNA synthesis, whereas high-intensity stimulation, particularly at 100 and 200 μA, progressively reduced the proportion of EdU-positive cells (Fig. 1F). A similar trend was observed in Ki67 immunostaining, in which the percentage of Ki67-positive cells was relatively maintained in the low- to moderate-current groups but decreased under higher current intensities, especially at 100 and 200 μA (Fig. 1G).

Collectively, these results indicate that microcurrent stimulation did not promote chondrocyte proliferation in 2D culture. The unchanged DNA content and CCK-8 values suggest that both total cell number and overall metabolic activity remained relatively stable across different stimulation intensities. In contrast, the reduced EdU incorporation and Ki67 expression in the 100- and 200-μA groups indicate that excessive microcurrent stimulation may partially suppress cell cycle activity and proliferative potential [24]. This finding is important for cartilage tissue engineering, as excessive electrical stimulation may impair chondrocyte biological activity even in the absence of obvious cytotoxicity.

### Microcurrent stimulation promoted the chondrogenic phenotype of chondrocytes in a dose-dependent manner

After establishing that microcurrent stimulation did not markedly promote chondrocyte proliferation, we next examined whether it modulated the chondrogenic phenotype of chondrocytes. In the 2D culture model, reverse transcription quantitative polymerase chain reaction (RT-qPCR) analysis at day 9 showed that microcurrent stimulation regulated the expression of cartilage-related genes in a dose-dependent manner. The messenger RNA (mRNA) expression levels of *collagen type II alpha 1 chain* (*Col2a1*), *SRY-box transcription factor 9* (*SOX9*), and *aggrecan* (*ACAN*) gradually increased from 0 to 50 μA, with the most pronounced up-regulation observed in the 50-μA group, and then declined when the stimulation intensity was further increased to 100 and 200 μA (Fig. 2A). These results suggest that low- to moderate-microcurrent stimulation enhanced the chondrogenic transcriptional profile of chondrocytes, whereas excessive stimulation attenuated this promotive effect.

**Fig. 2.**
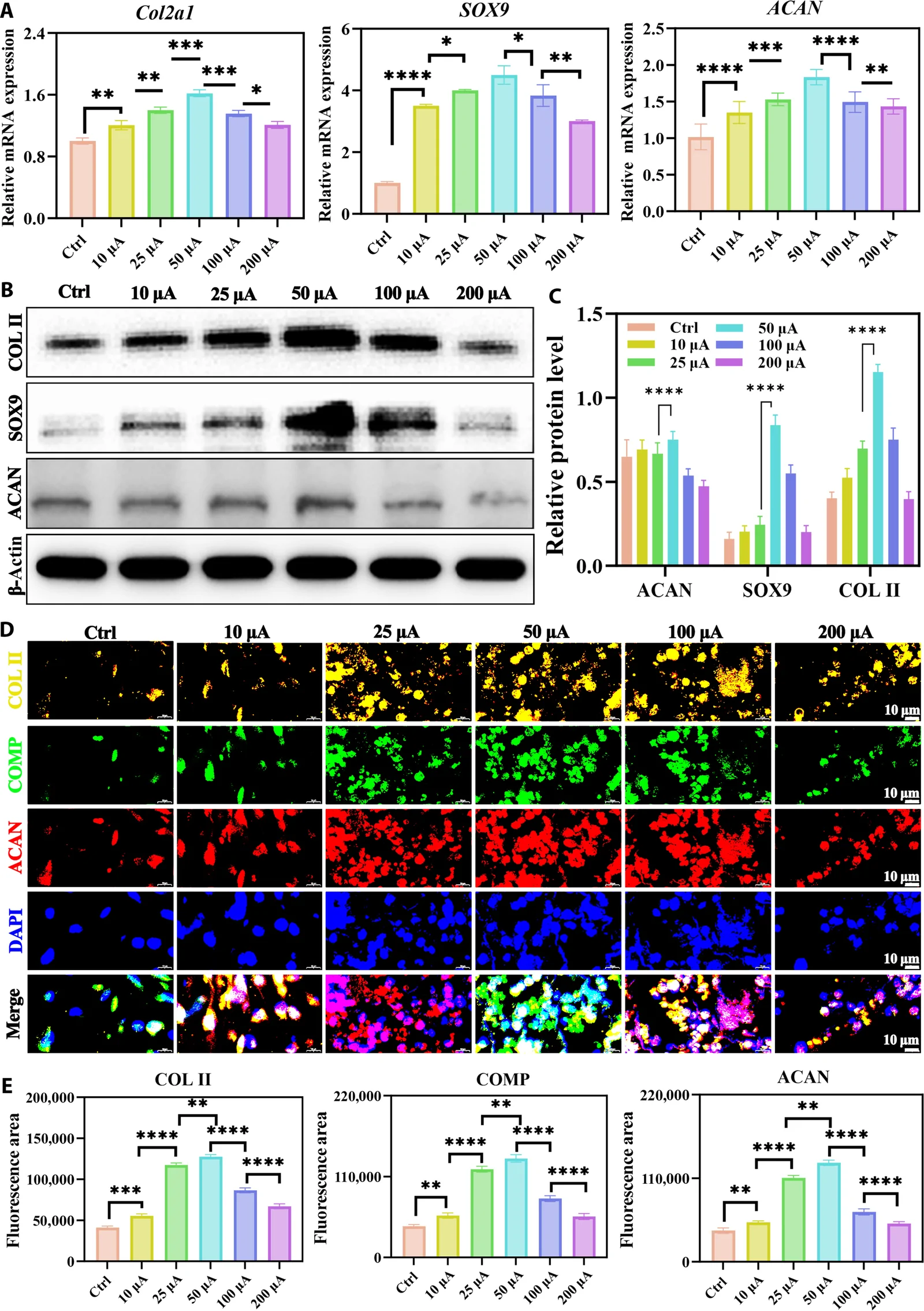
Dose-dependent effects of microcurrent stimulation on the chondrogenic phenotype of chondrocytes. (A) Relative messenger RNA (mRNA) expression levels of chondrogenic markers, namely, *collagen type II alpha 1 chain* (*Col2a1*), *SRY-box transcription factor 9* (*SOX9*), and *aggrecan* (*ACAN*), in chondrocytes from the control (Ctrl, 0 μA) and 10-, 25-, 50-, 100-, and 200-μA groups at day 9, as determined by reverse transcription quantitative polymerase chain reaction (RT-qPCR). (B) Representative Western blot images showing the protein expression levels of type II collagen (COL II), SOX9, and ACAN in chondrocytes from the Ctrl and 10-, 25-, 50-, 100-, and 200-μA groups at day 9, with β-actin used as the loading control. (C) Quantitative analysis of the relative protein expression levels of COL II, SOX9, and ACAN in each group based on the Western blot results shown in (B). (D) Representative immunofluorescence staining images of COL II, COMP, and ACAN in chondrocytes from the Ctrl and 10-, 25-, 50-, 100-, and 200-μA groups at day 9. (E) Quantitative analysis of the fluorescence-positive area of COL II, COMP, and ACAN in each group based on the immunofluorescence staining shown in (D). Data are presented as mean ± SD (*n* = 5). *P < 0.05; **P < 0.01; ***P < 0.001; ****P < 0.0001.

Consistent with the gene expression results, Western blot analysis at day 9 showed a similar dose-dependent pattern in cartilage-related protein expression. The protein levels of COL II, SOX9, and ACAN increased under low- to moderate-microcurrent stimulation and reached their highest levels in the 50-μA group. However, further increases in current intensity resulted in a gradual reduction in these protein levels (Fig. 2B). Quantitative analysis of the Western blot bands further confirmed this biphasic trend, showing progressive enhancement from 0 to 50 μA followed by attenuation from 50 to 200 μA (Fig. 2C).

Immunofluorescence staining further supported these findings. Compared with the control group, chondrocytes exposed to low- to moderate-microcurrent stimulation exhibited stronger fluorescence signals for COL II, cartilage oligomeric matrix protein (COMP), and ACAN, with the most evident enhancement observed in the 50-μA group (Fig. 2D). In contrast, the fluorescence signals weakened in the 100- and 200-μA groups. Quantitative analysis of the fluorescence-positive area or fluorescence intensity showed the same biphasic response, with cartilage-related marker expression increasing from 0 to 50 μA and then decreasing from 50 to 200 μA (Fig. 2E).

Together, these data indicate that microcurrent stimulation enhanced the matrix-producing phenotype of chondrocytes in a clear dose-dependent manner, even under monolayer culture conditions. Importantly, this promotive effect was not linearly proportional to current intensity. Instead, the response followed a biphasic pattern, in which low to moderate stimulation, particularly 50 μA, promoted the expression of cartilage-specific markers, whereas higher-intensity stimulation weakened this effect. This pattern suggests that microcurrent stimulation has a biologically meaningful therapeutic window, within which chondrogenic anabolic activity is enhanced without inducing unfavorable cellular responses.

To exclude the possibility that the observed biological differences were caused by nonspecific physical or chemical artifacts associated with electrical stimulation, the microenvironmental effects of prolonged stimulation were also examined. No obvious changes in temperature or pH were observed across groups from day 1 to day 9 (Fig. S2A and B), suggesting that gross thermal or acid–base disturbances were unlikely to account for the observed differences. In addition, reactive oxygen species (ROS) levels showed no significant differences among groups at day 9 (Fig. S2C), indicating that prolonged microcurrent stimulation did not induce detectable oxidative stress under the present experimental conditions. These findings suggest that the dose-dependent regulation of the chondrogenic phenotype was unlikely to result from nonspecific microenvironmental changes or electrochemical side effects but was more likely associated with the direct biological effects of microcurrent stimulation on chondrocytes.

### A 3D cartilage spheroid model revealed that optimal microcurrent stimulation improved spheroid morphology, matrix deposition, and cartilage-like tissue formation

Although 2D culture is useful for defining early cellular responses, it does not fully reproduce the native physicochemical and cell–matrix environment of cartilage [25]. Chondrocytes are highly sensitive to dimensional context, and their phenotype is generally better preserved in 3D systems [26]. Moreover, one important limitation of current electrical stimulation studies is that many observations are derived from 2D cultures, whereas evidence from 3D cartilage-like constructs remains relatively limited [27]. Therefore, to further validate the prochondrogenic effect of microcurrent stimulation under a more physiologically relevant condition, we established a 3D cartilage spheroid model.

Engineered cartilage spheroids were generated using 1 × 10^5^ auricular chondrocytes per spheroid and then subjected to microcurrent stimulation (Fig. 3A). Gross microscopic observation showed that microcurrent stimulation markedly influenced spheroid morphology at day 12 (Fig. 3B). The size of spheroids gradually increased as the stimulation intensity increased from 0 to 50 μA, with the largest and most regular spheroids observed in the 50-μA group. However, when the current intensity was further increased from 50 to 200 μA, spheroid size progressively decreased, and the spheroids became smaller and more contracted. Quantitative analysis further confirmed this biphasic morphological response, showing improved diameter and circularity in the low- to moderate-current groups, particularly at 50 μA, whereas high-intensity stimulation resulted in a reduced spheroid size and less favorable morphology at day 12 (Fig. 3C and Fig. S3). These findings suggest that optimal microcurrent stimulation supported spheroid growth and structural integrity, whereas excessive stimulation impaired spheroid morphological maturation.

**Fig. 3.**
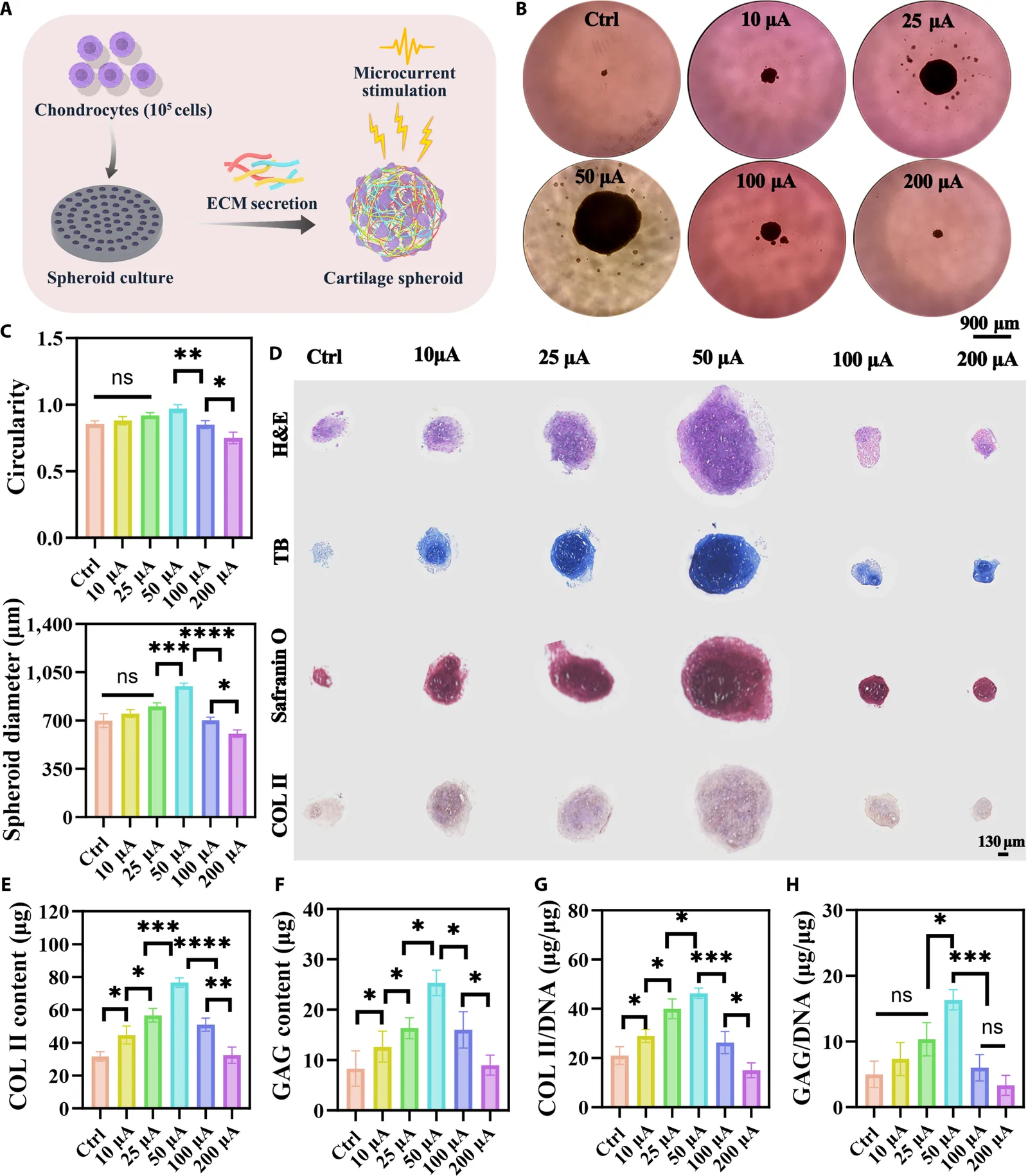
Evaluation of the effects of microcurrent stimulation on the regeneration of 3-dimensional (3D) cartilage spheroids. (A) Schematic illustration of the experimental design. Chondrocytes (1 × 10^5^ cells per spheroid) were seeded into spheroid molds and subsequently subjected to continuous direct microcurrent stimulation for 12 d. (B) Representative light microscopy images of cartilage spheroids in the control (Ctrl, 0 μA) and 10-, 25-, 50-, 100-, and 200-μA groups at day 12. (C) Quantitative analysis of spheroid circularity and diameter in the Ctrl and 10-, 25-, 50-, 100-, and 200-μA groups at day 12. (D) Representative hematoxylin and eosin (H&E), toluidine blue (TB), Safranin O, and type II collagen (COL II) immunohistochemical staining images of cartilage spheroids in the Ctrl and 10-, 25-, 50-, 100-, and 200-μA groups at day 12. Quantitative analysis of (E) COL II content and (F) glycosaminoglycan (GAG) content in the Ctrl and 10-, 25-, 50-, 100-, and 200-μA groups at day 12. Quantitative analysis of (G) COL II/DNA and (H) GAG/DNA levels in the Ctrl and 10-, 25-, 50-, 100-, and 200-μA groups at day 12. Data are presented as mean ± SD (*n* = 5). ns, not significant; *P < 0.05; **P < 0.01; ***P < 0.001; ****P < 0.0001.

The physicochemical and mechanical properties of cartilage spheroids were also improved by optimal microcurrent stimulation. Supplementary mechanical analysis showed that both the compressive modulus and dynamic storage modulus increased in the low- to moderate-current groups, with the highest values observed in the 50-μA group, whereas these parameters declined under excessive stimulation at day 12 (Fig. S4A and B). Similarly, indices related to tissue hydration and matrix retention, including water content and the wet-to-dry weight ratio, followed a comparable trend (Fig. S4C and D). These results suggest that optimal microcurrent stimulation improved not only spheroid morphology but also the functional quality and water-binding capacity of the engineered cartilage matrix.

Histological analysis further demonstrated that microcurrent stimulation strongly affected cartilage-like matrix formation. Hematoxylin and eosin (H&E), toluidine blue (TB), Safranin O, and immunohistochemical COL II stainings showed more abundant and homogeneous cartilage-like matrix deposition in the low- to moderate-current groups, particularly in the 50-μA group, whereas the 100- and 200-μA groups exhibited weaker metachromasia, reduced proteoglycan staining, and less organized tissue structure at day 12 (Fig. 3D). Enlarged H&E and Safranin O images further confirmed these differences, showing denser cartilage-like tissue organization and more intense proteoglycan-rich matrix staining in the 50-μA group, while high-intensity stimulation resulted in weaker matrix staining and inferior cartilage-like tissue formation (Fig. S4).

Consistently, biochemical quantification showed that the contents of COL II and GAG, as well as the normalized COL II/DNA and GAG/DNA values, progressively increased from 0 to 50 μA and subsequently decreased from 50 to 200 μA (Fig. 3E to H). These results indicate that 50-μA microcurrent stimulation most effectively promoted cartilage-specific ECM accumulation, whereas excessive stimulation attenuated matrix production despite the same 3D culture conditions.

Instead, the enhanced COL II and GAG deposition was more likely attributable to microcurrent-mediated regulation of the chondrogenic phenotype and matrix-producing activity. Taken together, these 3D results demonstrate that the beneficial effect of microcurrent stimulation observed in monolayer culture can be translated into enhanced cartilage-like tissue formation in a structured 3D microenvironment. Importantly, the response remained clearly dose dependent, with 50 μA representing the most favorable stimulation condition for spheroid morphology, ECM deposition, mechanical properties, and cartilage-like maturation. These findings also highlight the importance of evaluating electrical stimulation strategies in 3D cartilage models, where matrix context, spatial cell organization, and local signal distribution may substantially influence biological outcomes.

### Optimal microcurrent stimulation enhanced chondrogenic ECM production and preserved the hyaline cartilage-like phenotype of cartilage spheroids

To further characterize the quality and phenotypic stability of the matrix formed within 3D cartilage spheroids, immunofluorescence staining was performed at day 12. The expression of major cartilage ECM markers, including COL II, COMP, and ACAN, was markedly enhanced in the low- to moderate-microcurrent groups, with the strongest fluorescence signals observed in the 50-μA group (Fig. 4A). Quantitative analysis further confirmed this dose-dependent anabolic response, showing that the positive staining area or fluorescence intensity of COL II, COMP, and ACAN progressively increased from 0 to 50 μA and then declined when the current intensity was further increased to 100 and 200 μA (Fig. 4B). These results indicate that optimal microcurrent stimulation promoted the accumulation of cartilage-specific ECM components, whereas excessive stimulation attenuated this matrix-producing effect.

**Fig. 4.**
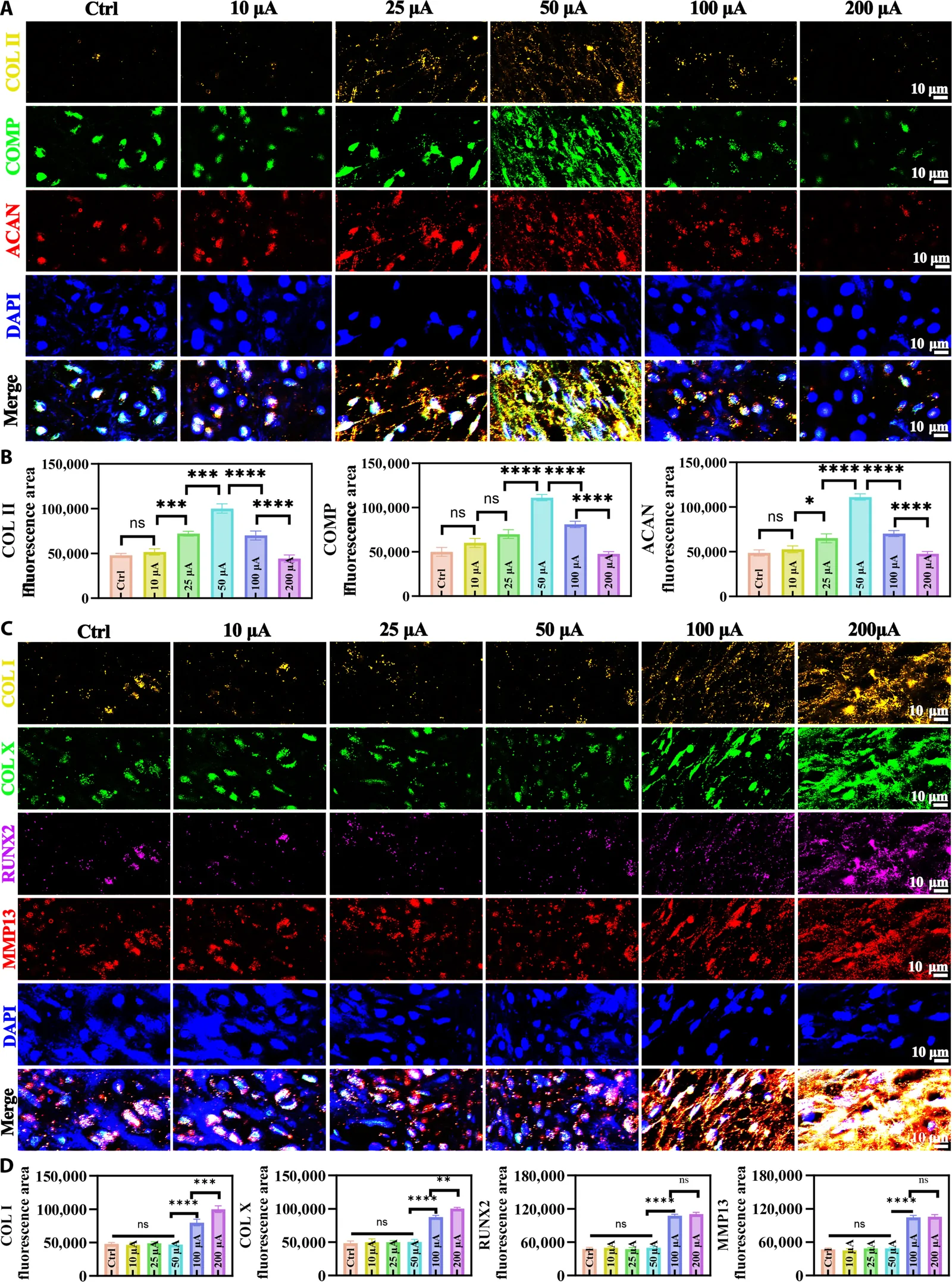
Immunofluorescence evaluation of the effects of microcurrent stimulation on chondrogenic extracellular matrix (ECM) production and dedifferentiation-related protein expression in 3-dimensional (3D) cartilage spheroids. (A) Representative immunofluorescence staining images of type II collagen (COL II), cartilage oligomeric matrix protein (COMP), and aggrecan (ACAN) in cartilage spheroids from the control (Ctrl, 0 μA) and 10-, 25-, 50-, 100-, and 200-μA groups at day 12. (B) Quantitative analysis of the fluorescence-positive area of COL II, COMP, and ACAN in each group based on the immunofluorescence staining shown in (A). (C) Representative immunofluorescence staining images of collagen type I (COL I), collagen type X (COL X), RUNX family transcription factor 2 (RUNX2), and matrix metallopeptidase 13 (MMP13) in cartilage spheroids from the Ctrl and 10-, 25-, 50-, 100-, and 200-μA groups at day 12. (D) Quantitative analysis of the fluorescence-positive area of COL I, COL X, RUNX2, and MMP13 in each group based on the immunofluorescence staining shown in (C). Data are presented as mean ± SD (*n* = 5). ns, not significant; *P < 0.05; **P < 0.01; ***P < 0.001; ****P < 0.0001.

In contrast, immunofluorescence staining of dedifferentiation-, hypertrophy-, and matrix-degradation-related markers showed an opposite tendency. The expression of collagen type I (COL I), collagen type X (COL X), RUNX family transcription factor 2 (RUNX2), and matrix metallopeptidase 13 (MMP13) became more prominent under high-intensity stimulation, particularly in the 100- and 200-μA groups (Fig. 4C). Quantitative analysis confirmed that these unfavorable markers remained relatively low in the low- to moderate-current groups but were markedly increased under excessive microcurrent stimulation (Fig. 4D). Since COL I is associated with fibrocartilaginous or dedifferentiated matrix formation, COL X and RUNX2 are closely related to chondrocyte hypertrophy, and MMP13 is a key matrix-degrading enzyme, the increased expression of these markers suggests that excessive microcurrent stimulation impaired phenotypic stability and promoted a less favorable cartilage-forming state.

Together, these findings demonstrate that the effect of microcurrent stimulation on cartilage spheroids was not limited to increasing the amount of ECM deposition. More importantly, optimal stimulation, especially at 50 μA, primarily preserved the chondrogenic phenotype and limited dedifferentiation, rather than inducing de novo differentiation, while enhancing cartilage-specific ECM components and suppressing hypertrophy- and degradation-associated responses. This balance between enhanced anabolic matrix production and reduced unfavorable phenotypic deviation is critical for generating stable hyaline cartilage-like tissue [28].

Combined with the morphological, biochemical, mechanical, and hydration-related results described above, the immunofluorescence data further support the existence of an optimal microcurrent stimulation window for engineered cartilage maturation [29]. Within this window, microcurrent stimulation promoted a coordinated cartilage-forming response characterized by improved spheroid morphology, increased COL II- and GAG-rich matrix accumulation, enhanced matrix-related functional properties, and preservation of a hyaline cartilage-like phenotype. By contrast, stimulation beyond this window weakened cartilage-specific ECM production and shifted spheroids toward a more fibrocartilaginous, hypertrophic, and catabolic phenotype. Therefore, 50-μA microcurrent stimulation appears to provide a favorable bioelectrical cue for promoting not only ECM synthesis but also phenotypic stability and functional maturation of engineered cartilage spheroids.

### Excessive microcurrent stimulation induced catabolic, inflammatory, and apoptosis-related responses in cartilage spheroids

Because matrix formation declined under high-intensity stimulation, we next investigated whether excessive microcurrent stimulation induced a stress-associated phenotype in cartilage spheroids. Immunofluorescence staining revealed that the expression of catabolic and inflammatory markers, including a disintegrin and metalloproteinase with thrombospondin motifs 5 (ADAMTS5), inducible nitric oxide synthase (iNOS), and cyclooxygenase-2 (COX-2), was elevated under excessive stimulation, particularly in the 100- and 200-μA groups at day 12 (Fig. 5A). Quantitative analysis further confirmed that the positive staining area of these markers remained relatively low in the 0- to 50-μA groups but increased markedly when the stimulation intensity exceeded the optimal range (Fig. 5B). These findings indicate that excessive microcurrent stimulation shifted chondrocytes from an anabolic matrix-producing state toward a catabolic and inflammation-associated phenotype, which is unfavorable for stable cartilage matrix accumulation.

**Fig. 5.**
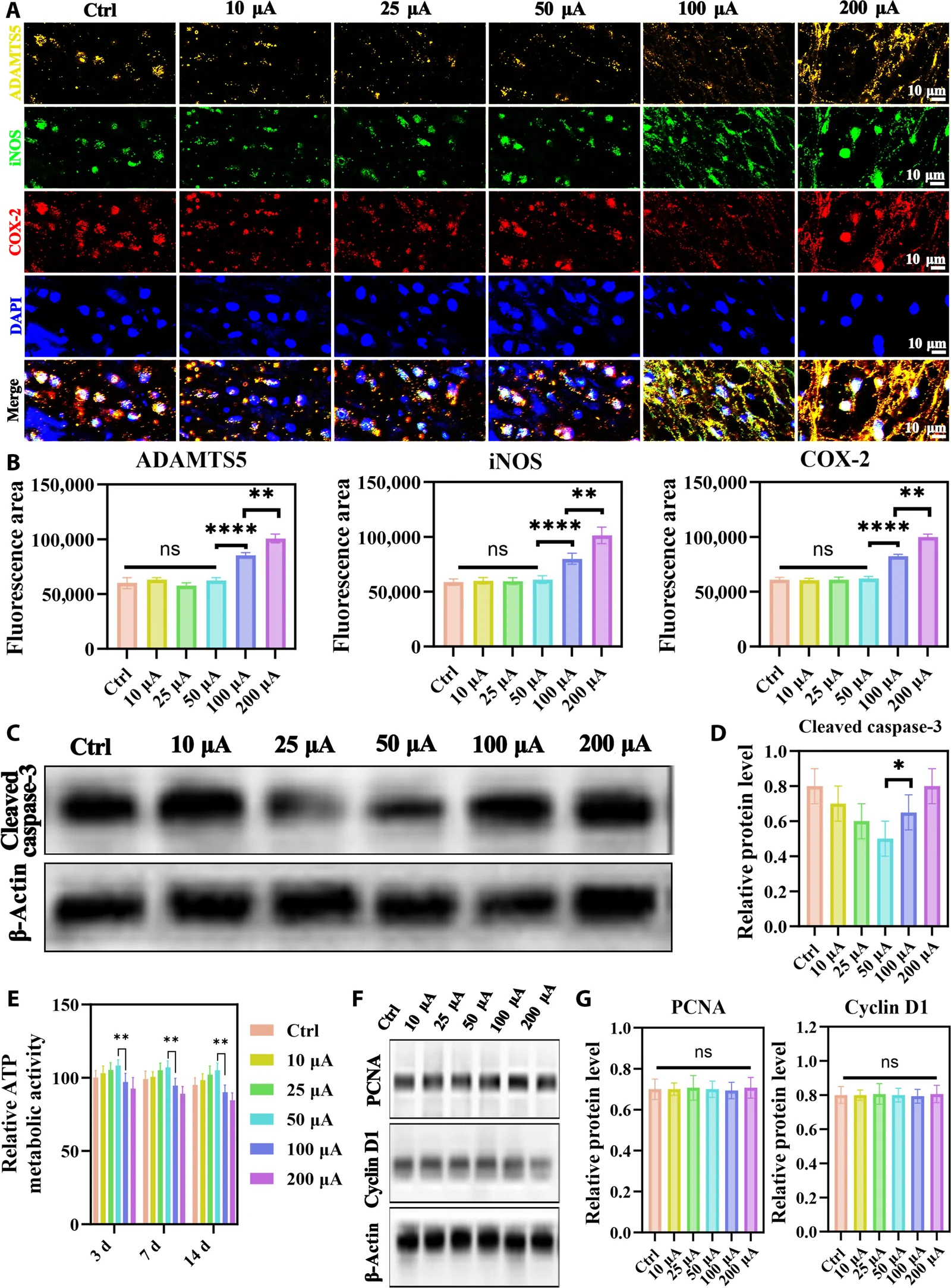
Evaluation of catabolic, apoptotic, metabolic, and proliferation-related responses in cartilage spheroids under microcurrent stimulation. (A) Representative immunofluorescence staining images of a disintegrin and metalloproteinase with thrombospondin motifs 5 (ADAMTS5), inducible nitric oxide synthase (iNOS), and cyclooxygenase-2 (COX-2) in cartilage spheroids from the control (Ctrl, 0 μA) and 10-, 25-, 50-, 100-, and 200-μA groups at day 12. (B) Quantitative analysis of the fluorescence-positive area of ADAMTS5, iNOS, and COX-2 in each group based on the immunofluorescence staining shown in (A). (C) Representative Western blot images showing the protein expression levels of cleaved caspase-3 in cartilage spheroids from the Ctrl and 10-, 25-, 50-, 100-, and 200-μA groups at day 12, with β-actin used as the loading control. (D) Quantitative analysis of the relative protein expression levels of cleaved caspase-3 in each group based on the Western blot results shown in (C). (E) Adenosine triphosphate (ATP)-based metabolic activity, as measured by the CellTiter-Glo assay, in cartilage spheroids from the Ctrl and 10-, 25-, 50-, 100-, and 200-μA groups at days 3, 7, and 14. (F) Representative Western blot images showing the protein expression levels of proliferating cell nuclear antigen (PCNA) and cyclin D1 in cartilage spheroids from the Ctrl and 10-, 25-, 50-, 100-, and 200-μA groups at day 12, with β-actin used as the loading control. (G) Quantitative analysis of the relative protein expression levels of PCNA and cyclin D1 in each group based on the Western blot results shown in (F). Data are presented as mean ± SD (*n* = 5). ns, not significant; **P < 0.01; ****P < 0.0001.

To determine whether the enhanced matrix formation observed under low to moderate stimulation was associated with increased proliferative expansion, proliferation-related genes, including *marker of proliferation Ki67* (*Mki67*), *proliferating cell nuclear antigen* (*Pcna*), and *cyclin D1* (*Ccnd1*), were further examined in cartilage spheroids. These genes did not exhibit a consistent promotive response across stimulation groups from day 3 to day 14 (Fig. S6), suggesting that the improved cartilage-like tissue formation in the low- to moderate-current groups was not primarily driven by increased cell proliferation.

Live/Dead staining at days 3 and 7 showed abundant viable cells and few dead cells in all groups, with no obvious differences among the different stimulation intensities (Fig. S7). In addition, all groups exhibited increased viable cell coverage over time, indicating good cell survival and growth within the 3D spheroids during culture. Terminal deoxynucleotidyl transferase dUTP nick-end labeling (TUNEL) staining showed a similar pattern to the Live/Dead results, with low levels of apoptotic cells observed across all groups at both day 3 and day 7 (Fig. S8A and B). Quantitative analysis of TUNEL-positive cells further confirmed that there were no significant differences in apoptosis among groups at either time point (Fig. S8C and D). These findings indicate that the beneficial effect of low- to moderate-microcurrent stimulation on cartilage-like matrix formation was unlikely to be explained by enhanced cell proliferation, improved survival, or reduced apoptosis.

Cleaved caspase-3 expression showed a biphasic response to microcurrent stimulation (Fig. 5C and D). Its level gradually decreased from 0 to 50 μA, suggesting that optimal microcurrent stimulation may reduce basal apoptosis-associated molecular stress and support cellular homeostasis within cartilage spheroids. However, when the stimulation intensity was further increased from 50 to 200 μA, cleaved caspase-3 expression progressively increased, indicating that excessive microcurrent stimulation may activate apoptosis-related signaling beyond the optimal stimulation window. Notably, because Live/Dead and TUNEL staining did not show obvious differences among groups, the increase in cleaved caspase-3 should be interpreted as evidence of apoptosis-associated molecular stress rather than extensive cell death [30]. This biphasic pattern is consistent with the dose-dependent changes in cartilage matrix deposition, suggesting that the reduced ECM formation under high-intensity stimulation may be partly associated with activation of apoptosis-related molecular pathways, even in the absence of overt cytotoxicity.

The bcl-2-associated X protein (Bax)/B-cell lymphoma 2 (Bcl-2) axis further supported this interpretation. Bax is a pro-apoptotic member of the Bcl-2 family that promotes mitochondrial apoptotic signaling, whereas Bcl-2 is an anti-apoptotic protein that helps maintain mitochondrial integrity and cell survival. Therefore, the balance between Bax and Bcl-2 is commonly used to evaluate the tendency of cells toward either survival or apoptosis [31,32]. In the present study, Bax expression remained relatively low in the low-current groups but increased under high-intensity stimulation, whereas Bcl-2 showed the opposite trend, being relatively preserved in the 0- to 50-μA groups and decreased in the 100- to 200-μA groups (Fig. S9A to C). As a result, the Bax/Bcl-2 ratio was lowest in the optimal microcurrent groups and highest in the 200-μA group (Fig. S9D), indicating a shift toward a pro-apoptotic molecular state under excessive stimulation. This pattern was highly consistent with the increase in cleaved caspase-3 and the reduction in ECM formation under high-current conditions.

In contrast, adenosine triphosphate (ATP)-based metabolic activity measured by CellTiter-Glo remained relatively stable under low to moderate stimulation but showed an apparent decreasing trend in the 100- and 200-μA groups (Fig. 5E). Since CellTiter-Glo reflects ATP-dependent metabolic activity in 3D spheroids [33], this result suggests that excessive microcurrent stimulation may compromise cellular metabolic fitness, even though obvious cytotoxicity was not detected by Live/Dead staining. Meanwhile, PCNA and cyclin D1 protein levels were largely unchanged across groups (Fig. 5F and G). PCNA is associated with DNA replication and cell cycle progression, whereas cyclin D1 regulates the G1/S transition of the cell cycle [34]. The absence of marked changes in these proliferation-related proteins further indicates that the dose-dependent decline in cartilage matrix formation under high-intensity stimulation was not primarily caused by altered proliferative activity.

Together, these results suggest that excessive microcurrent stimulation did not simply reduce cartilage formation by decreasing cell number or inducing overt cell death. Instead, high-current stimulation appeared to disturb cartilage spheroid homeostasis through dysregulated cellular signaling, including increased catabolic and inflammatory marker expression, reduced ATP-related metabolic activity, and activation of apoptosis-associated signaling. By contrast, 50-μA stimulation maintained a more favorable balance, characterized by low catabolic and inflammatory responses, reduced apoptosis-associated molecular stress, preserved metabolic activity, and enhanced cartilage-specific ECM deposition. These findings further support the existence of an optimal microcurrent stimulation window, beyond which the biological response shifts from anabolic cartilage regeneration toward stress-associated matrix deterioration.

### Optimal microcurrent stimulation activated Ca^2+^/calcineurin/NFAT signaling, whereas excessive stimulation failed to generate effective prochondrogenic signaling

To elucidate the mechanism underlying the dose-dependent effect of microcurrent stimulation, we focused on intracellular Ca^2+^ signaling and downstream NFAT activation. Fluo-4 acetoxymethyl ester (Fluo-4 AM) calcium imaging showed distinct Ca^2+^ response patterns among the experimental groups (Fig. 6A). Compared with the control (Ctrl) group, the 50-μA group exhibited a robust but relatively coordinated Ca^2+^ response, indicating that moderate microcurrent stimulation effectively activated intracellular Ca^2+^ signaling in cartilage spheroids. In contrast, the 200-μA group displayed a more dysregulated Ca^2+^ response pattern, suggesting that excessive stimulation did not simply amplify the beneficial signal generated by moderate stimulation.

**Fig. 6.**
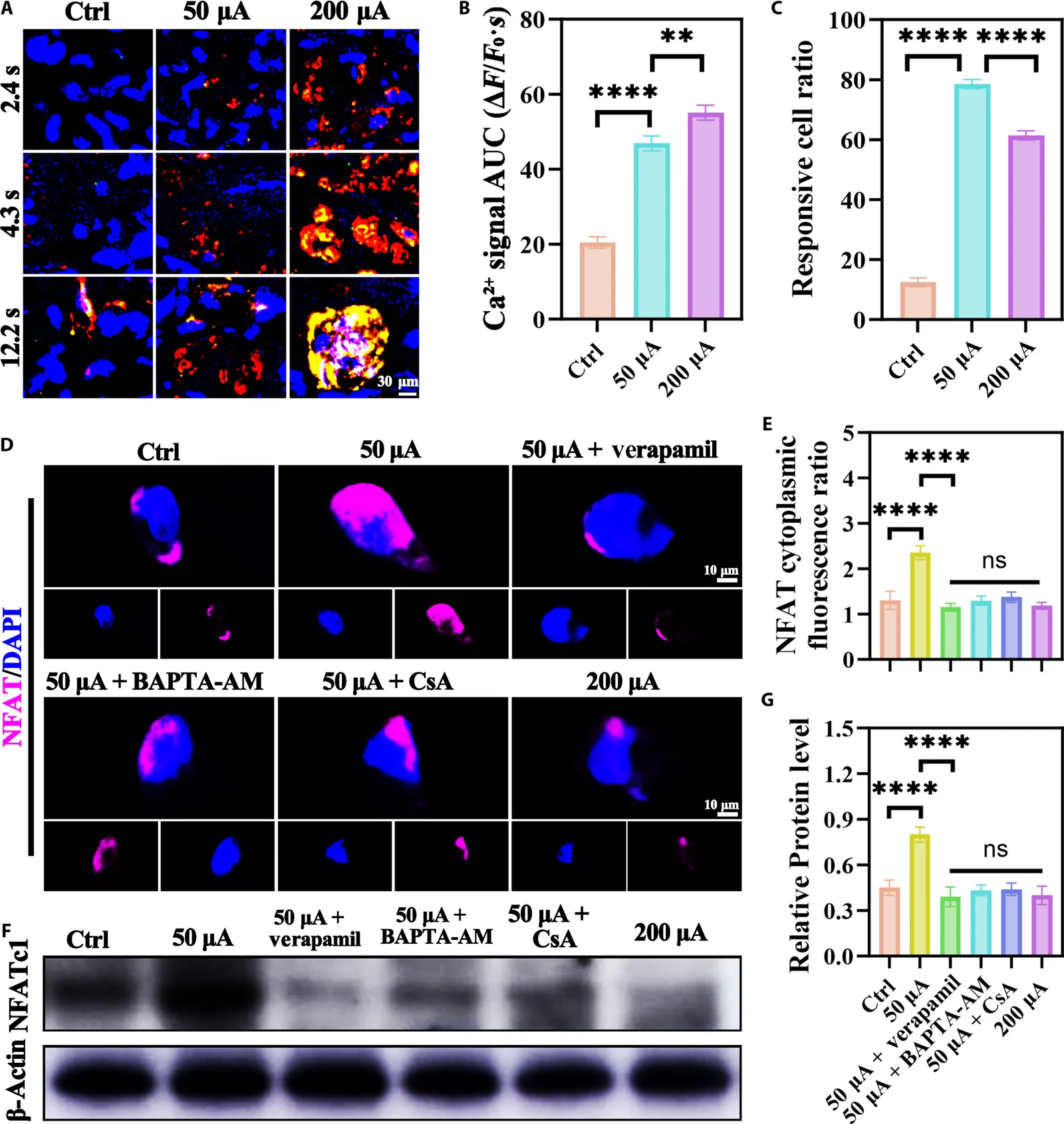
Microcurrent stimulation regulates intracellular Ca^2+^ signaling and nuclear factor of activated T cells (NFAT) activation in cartilage spheroids. (A) Representative fluo-4 acetoxymethyl ester (Fluo-4 AM) calcium imaging snapshots of cartilage spheroids in the 0-, 50-, and 200-μA groups, captured at their respective peak-response time points (2.4, 4.3, and 12.2 s). Quantitative analysis of (B) the Ca^2+^ signal area under the curve (AUC) (Δ*F*/*F*_0_·*s*) and (C) the responsive cell ratio in the 0-, 50-, and 200-μA groups during the first 60 s after stimulation onset. (D) Representative immunofluorescence staining images of NFAT and 4′,6-diamidino-2-phenylindole (DAPI) in cartilage spheroids from the 0 μA, 50 μA, 50 μA + verapamil, 50 μA + BAPTA-AM, 50 μA + CsA, and 200 μA groups at day 12. BAPTA-AM, 1,2-bis(2-aminophenoxy)ethane-*N*,*N*,*N*′,*N*′-tetraacetic acid acetoxymethyl ester; CsA, cyclosporin A. (E) Quantitative analysis of the NFAT nuclear/cytoplasmic fluorescence ratio in each group. (F) Representative Western blot images showing the protein expression levels of NFATc1 in cartilage spheroids from the 0 μA, 50 μA, 50 μA + verapamil, 50 μA + BAPTA-AM, 50 μA + CsA, and 200 μA groups at day 12, with β-actin used as the loading control. (G) Quantitative analysis of the relative protein expression levels of NFATc1 in each group based on the Western blot results shown in (F). Data are presented as mean ± SD (*n* = 5). ns, not significant; **P < 0.01; ****P < 0.0001.

Quantitative analysis further clarified this difference. The Ca^2+^ signal area under the curve (AUC) was markedly increased in the 50-μA group compared with that in the Ctrl group and was further elevated in the 200-μA group (Fig. 6B). This indicates that high-intensity stimulation produced a greater overall Ca^2+^ signal burden. However, the responsive cell ratio was markedly increased in the 50-μA group compared with that in the Ctrl group, whereas it was markedly reduced in the 200-μA group compared with that in the 50-μA group (Fig. 6C). These results suggest that 50-μA stimulation recruited a larger proportion of cells into an effective and coordinated Ca^2+^ response, whereas 200-μA stimulation generated excessive Ca^2+^ accumulation in fewer responsive cells or induced an abnormal Ca^2+^ response pattern. Thus, the biological outcome of Ca^2+^ signaling depended not only on signal magnitude but also on the coordination and cellular distribution of the response.

To further determine whether Ca^2+^ influx and downstream calcineurin/NFAT signaling were required for microcurrent-induced prochondrogenic signaling, pharmacological inhibitors were introduced. Verapamil, a voltage-gated calcium channel blocker, was used to evaluate whether calcium entry through calcium channels contributed to the microcurrent-induced response. 1,2-Bis(2-aminophenoxy)ethane-*N*,*N*,*N*′,*N*′-tetraacetic acid acetoxymethyl ester (BAPTA-AM), an intracellular Ca^2+^ chelator, was used to buffer cytosolic Ca^2+^ and determine whether intracellular Ca^2+^ elevation was necessary for downstream signaling. Cyclosporin A (CsA), a calcineurin inhibitor, was used to block calcineurin-dependent NFAT activation [35]. Therefore, the 50 μA + verapamil, 50 μA + BAPTA-AM, and 50 μA + CsA groups were designed to dissect the signaling sequence from Ca^2+^ influx to intracellular Ca^2+^ elevation and subsequent calcineurin/NFAT activation. Immunofluorescence staining demonstrated that NFAT nuclear translocation was markedly enhanced in the 50-μA group compared with that in the 0-μA group (Fig. 6D and E). This effect was markedly attenuated by verapamil, BAPTA-AM, and CsA, indicating that microcurrent-induced NFAT activation depended on Ca^2+^ entry, intracellular Ca^2+^ signaling, and calcineurin activity. Further validation using genetic approaches may strengthen this mechanistic conclusion. In contrast, the 200-μA group did not show a similarly effective NFAT nuclear translocation pattern, despite its higher Ca^2+^ signal AUC. This finding suggests that excessive Ca^2+^ signaling was not efficiently converted into productive NFAT activation. Western blot analysis of NFAT further supported the immunofluorescence results, showing that optimal microcurrent stimulation enhanced NFAT-related signaling, whereas inhibition of Ca^2+^/calcineurin signaling weakened this effect (Fig. 6F and G).

Quantitative analysis of the percentage of cells with nuclear NFAT localization further confirmed this conclusion. The 50-μA group showed a markedly higher proportion of cells with nuclear NFAT localization than the 0-μA group, whereas treatment with verapamil, BAPTA-AM, or CsA markedly reduced this proportion (Fig. S10). Moreover, the 200-μA group failed to maintain the same level of NFAT nuclear localization as the 50-μA group, indicating that excessive stimulation did not generate an optimal NFAT-dependent prochondrogenic signaling response.

These results support a model in which moderate microcurrent stimulation induces an appropriate and coordinated intracellular Ca^2+^ response, which subsequently activates the Ca^2+^/calmodulin/calcineurin/NFAT axis and promotes a signaling environment favorable for chondrogenic matrix production [36]. However, the specific upstream calcium channels responsible for initiating microcurrent-induced Ca^2+^ responses, potentially including mechanosensitive Piezo channels, remain to be further elucidated.

In contrast, excessive stimulation produced a disproportionate Ca^2+^ response characterized by a higher total Ca^2+^ signal AUC but a reduced responsive cell ratio and insufficient NFAT nuclear translocation [37]. This suggests that NFAT activation depends on coordinated Ca^2+^ signaling rather than total Ca^2+^ magnitude alone; the higher AUC but lower responsive cell ratio at 200 μA may reflect dysregulated Ca^2+^ accumulation that is inefficiently translated into calcineurin/NFAT activation. Under this condition, cartilage spheroids showed increased catabolic and inflammatory marker expression, activation of apoptosis-associated molecular signals, and reduced ECM deposition [38]. Therefore, the impaired matrix formation in the high-current groups was likely related not to a lack of Ca^2+^ signaling but to dysregulated Ca^2+^ signaling that failed to support coordinated NFAT-mediated anabolic responses.

Taken together, these findings demonstrate that the beneficial effect of microcurrent stimulation cannot be understood simply as “more current is better”. Instead, microcurrent stimulation appears to operate within a functional bioelectrical window. Within this window, represented by 50 μA in the present study, electrical input is effectively converted into coordinated Ca^2+^/calcineurin/NFAT signaling and chondrogenic matrix formation. Beyond this window, excessive stimulation generates dysregulated Ca^2+^ responses, weakens effective NFAT activation, and shifts cartilage spheroids toward stress-associated matrix deterioration rather than anabolic cartilage regeneration. Although our study demonstrated enhanced cartilage-specific ECM formation through histological staining, biochemical quantification, and mechanical characterization, further ultrastructural analyses, such as scanning electron microscopy or second-harmonic generation imaging, are needed to elucidate the detailed organization and remodeling of ECM architecture following microcurrent stimulation.

### Proposed mechanism of microcurrent-regulated cartilage matrix formation

Based on the above findings, a schematic mechanism was proposed to summarize the beneficial and detrimental responses to dose-dependent microcurrent stimulation (Fig. 7A). Under the 0-μA condition, chondrocytes maintained a basal level of Ca^2+^ signaling, limited NFAT nuclear translocation, and baseline ECM synthesis. Under optimal microcurrent stimulation, particularly at 50 μA, a moderate and coordinated Ca^2+^ response was induced, which activated the Ca^2+^/calcineurin/NFAT signaling axis, promoted NFAT nuclear translocation, and supported SOX9-centered chondrogenic transcription and the expression of matrix-related markers, including COL II, ACAN, and COMP. This signaling pattern favored cartilage-specific ECM accumulation and helped maintain a stable hyaline cartilage-like phenotype.

**Fig. 7.**
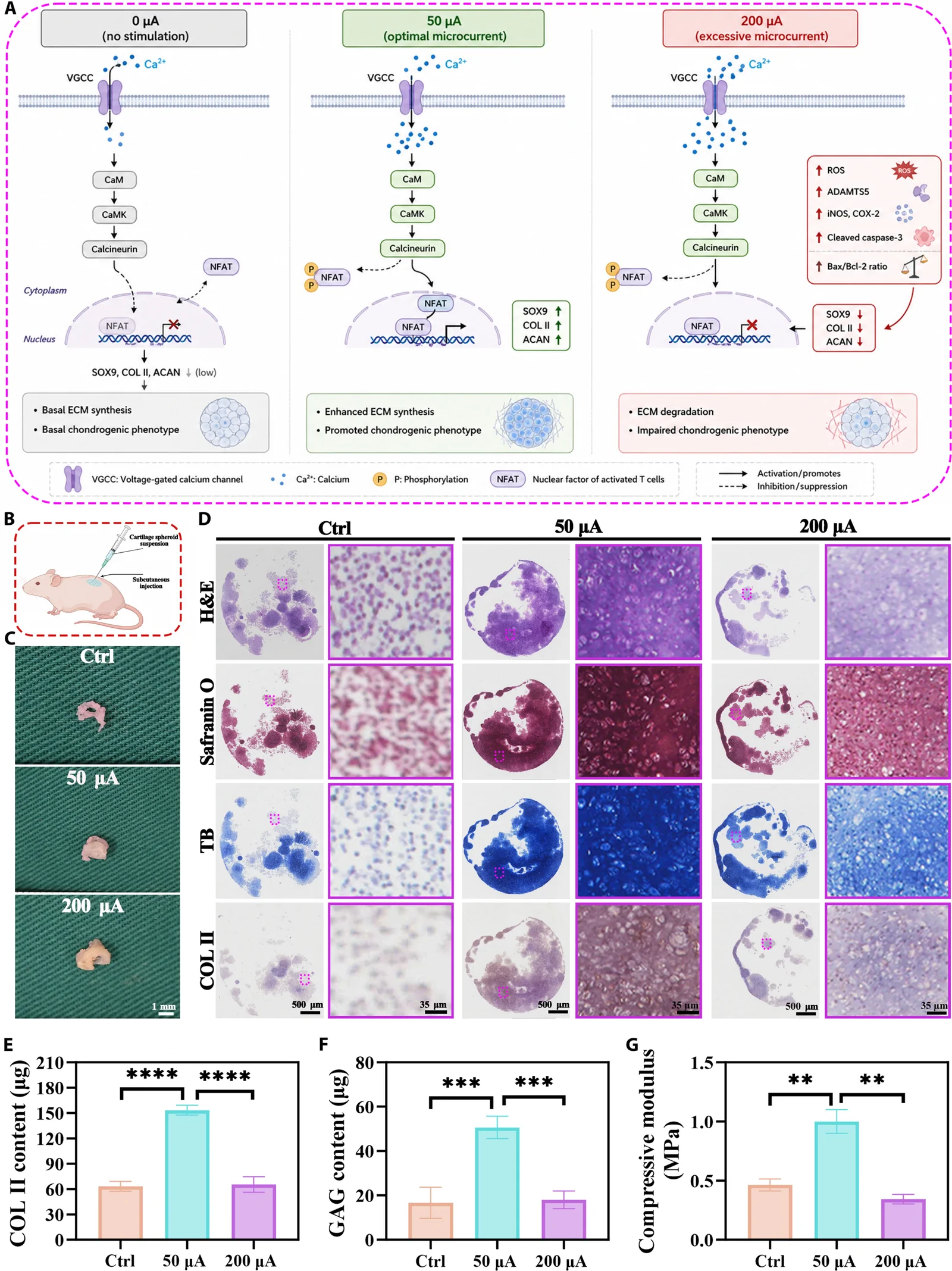
In vivo validation of the effects of microcurrent stimulation on cartilage matrix formation after subcutaneous implantation. (A) Schematic illustration summarizing the beneficial and detrimental responses to microcurrent stimulation in engineered chondrocyte spheroids, highlighting coordinated Ca^2+^/calcineurin/nuclear factor of activated T cells (NFAT)-mediated anabolic signaling at 50 μA and dysregulated stress-associated responses at 200 μA. (B) Schematic illustration of subcutaneous injection of cartilage spheroid suspensions from the 0-, 50-, and 200-μA groups into nude mice. (C) Representative gross images of samples after 2 weeks of subcutaneous implantation in nude mice. (D) Representative hematoxylin and eosin (H&E), toluidine blue (TB), Safranin O, and type II collagen (COL II) immunohistochemical staining images of samples from the 0-, 50-, and 200-μA groups after 2 weeks of subcutaneous implantation in nude mice. The right panels show enlarged views of the corresponding regions in the left panels. Quantitative analysis of (E) COL II content, (F) glycosaminoglycan (GAG) content, and (G) compressive modulus of samples from the 0-, 50-, and 200-μA groups after 2 weeks of subcutaneous implantation in nude mice. Data are presented as mean ± SD (*n* = 5). **P < 0.01; ***P < 0.001; ****P < 0.0001.

In contrast, excessive microcurrent stimulation, represented by 200 μA, generated a dysregulated Ca^2+^ response rather than a more effective prochondrogenic signal. Although the overall Ca^2+^ signal burden was increased, the responsive cell ratio and NFAT nuclear translocation were not correspondingly improved, indicating that excessive stimulation failed to efficiently convert Ca^2+^ signaling into productive NFAT-mediated anabolic activity [27]. In parallel, high-intensity stimulation was associated with increased expression of catabolic and inflammatory markers, including ADAMTS5, iNOS, and COX-2, as well as apoptosis-associated molecular changes, such as elevated cleaved caspase-3, increased Bax expression, reduced Bcl-2 expression, and a higher Bax/Bcl-2 ratio. Notably, temperature, pH, and ROS levels did not show obvious differences among groups, suggesting that the impaired cartilage matrix formation under excessive stimulation was unlikely to be caused by nonspecific thermal, acid–base, or oxidative microenvironmental changes. Instead, it was more likely related to dysregulated bioelectrical signaling and stress-associated cellular responses.

This proposed mechanism integrates the major findings of the present study into a coherent model. Microcurrent stimulation did not markedly promote chondrocyte proliferation, as indicated by stable DNA content, CCK-8 activity, proliferation-related gene expression, and proliferation-associated protein levels. This is important because excessive proliferation is not necessarily beneficial for cartilage regeneration and may compromise chondrocyte phenotype stability [8]. Within an appropriate stimulation window, however, microcurrent stimulation enhanced cartilage matrix formation primarily through functional and phenotypic regulation rather than proliferative expansion.

Therefore, the regenerative effect of microcurrent stimulation should not be interpreted as a simple intensity-dependent enhancement in which stronger stimulation produces better outcomes. Instead, the present data support the existence of a functional bioelectrical window. Within this window, 50-μA stimulation effectively coordinated Ca^2+^/calcineurin/NFAT signaling, enhanced cartilage-specific ECM production, and preserved a favorable chondrogenic phenotype. Beyond this window, excessive stimulation disturbed cellular signaling homeostasis; activated catabolic, inflammatory, and apoptosis-associated molecular responses; and ultimately weakened cartilage matrix accumulation. Defining this optimal current range is therefore essential for applying microcurrent stimulation in cartilage tissue engineering.

### In vivo subcutaneous implantation validated the superior cartilage-forming potential of 50-μA-preconditioned spheroids

To determine whether the in vitro findings could be translated into improved cartilage formation in vivo, cartilage spheroid suspensions from the 0-, 50-, and 200-μA groups were injected subcutaneously into nude mice and harvested after 2 weeks (Fig. 7B). Gross observation showed that the 50-μA group formed more robust and cartilage-like tissue than the control group, whereas the 200-μA group exhibited inferior tissue formation and less favorable gross morphology (Fig. 7C).

Histological and immunohistochemical analyses were consistent with the in vitro results. H&E, Safranin O, and TB staining demonstrated more abundant and better-organized cartilage-like matrix in the 50-μA group, whereas the 200-μA group showed weaker matrix staining and poorer tissue organization (Fig. 7D). COL II immunohistochemistry further confirmed stronger cartilage-specific matrix deposition in the 50-μA-preconditioned group. Quantitative analyses showed that COL II content, GAG content, and compressive modulus were all highest in the 50-μA group, while these parameters were reduced in the 200-μA group (Fig. 7E to G). These findings indicate that optimal microcurrent preconditioning improved not only histological cartilage-like matrix formation but also the biochemical and mechanical quality of the regenerated tissue.

These in vivo results strongly support the translational relevance of the in vitro observations. Importantly, the superior cartilage-forming performance of the 50-μA group after implantation suggests that microcurrent preconditioning did not merely induce transient molecular changes during in vitro culture. Instead, it appeared to establish a more favorable biological state in cartilage spheroids, characterized by enhanced chondrogenic matrix-producing capacity and better phenotypic stability, which could be retained after transplantation and translated into improved tissue formation in vivo.

This finding is particularly relevant for injectable cartilage tissue engineering. Because spheroids or microtissues can be delivered in a minimally invasive manner, optimizing their biological quality before implantation may be an effective strategy to improve subsequent cartilage regeneration [39]. The present results suggest that 50-μA microcurrent preconditioning may serve as a practical bioelectrical maturation strategy for enhancing the regenerative potential of engineered cartilage spheroids, although stimulation duration, delivery approaches, and long-term safety require further optimization before clinical translation.

## Conclusion

In conclusion, this study demonstrates that microcurrent stimulation regulates cartilage matrix formation in a dose-dependent manner rather than acting as a uniformly beneficial stimulus. Microcurrent stimulation did not markedly enhance chondrocyte proliferation or general metabolic activity in 2D culture, but it promoted chondrogenic marker expression within an optimal intensity range. In engineered cartilage spheroids, 50-μA stimulation most effectively improved spheroid morphology, cartilage-specific ECM deposition, mechanical properties, and hyaline cartilage-like phenotypic stability, whereas higher intensities impaired matrix formation and induced catabolic, inflammatory, and apoptosis-associated molecular responses without overt cytotoxicity or nonspecific microenvironmental changes. Mechanistically, optimal stimulation activated coordinated Ca^2+^/calcineurin/NFAT signaling, while excessive stimulation produced dysregulated Ca^2+^ responses that failed to support efficient NFAT-mediated prochondrogenic signaling. The superior cartilage-forming capacity of 50-μA-preconditioned spheroids after subcutaneous implantation further confirmed the translational potential of this optimized stimulation strategy. Overall, these findings identify 50 μA as an effective bioelectrical stimulation window for cartilage spheroid maturation and highlight the importance of precise current control in microcurrent-assisted cartilage tissue engineering.

## Materials and Methods

### Isolation and culture of auricular chondrocytes

All animal procedures were reviewed and approved by the Institutional Animal Care and Use Committee of Shanghai Pulmonary Hospital (No. K21-355Y) and were performed in accordance with the institutional guidelines for the care and use of laboratory animals. Primary auricular chondrocytes were isolated from Sprague-Dawley rats (3 to 4 weeks old; Shanghai Yunde Biotechnology Co., Ltd., China) under sterile conditions. Briefly, auricular cartilage was harvested, washed 3 times in phosphate-buffered saline (PBS; Sigma-Aldrich, USA) supplemented with 1% penicillin–streptomycin (Sigma-Aldrich, USA), and cut into approximately 1-mm^3^ fragments. Tissues were digested with 0.2% collagenase II (Sigma-Aldrich, USA) in Dulbecco’s modified Eagle’s medium/F12 (DMEM/F12; Sigma-Aldrich, USA) at 37 °C for 8 h with gentle shaking. The digested suspension was filtered through a 70-μm cell strainer and centrifuged at 300 × *g* for 5 min. Cells were resuspended in complete medium consisting of DMEM/F12 supplemented with 10% fetal bovine serum (Sigma-Aldrich, USA) and 1% penicillin–streptomycin and cultured in a humidified incubator at 37 °C with 5% CO_2_. Passage 2 cells were used for all experiments.

### Microcurrent stimulation setup and experimental grouping

Microcurrent stimulation was delivered using a programmable constant-current stimulator (ECM 830, Harvard Apparatus, Holliston, MA, USA) connected to culture-compatible platinum wire electrodes (Sigma-Aldrich, USA). The electrodes were sterilized before use and positioned in parallel on opposite sides of each well to provide a standardized electrical configuration across experimental groups. The distance between the 2 electrodes was maintained at approximately 5 mm for 2D cultures in 96-well plates and 5 mm for spheroid cultures, depending on the well format. The immersed electrode length was approximately 5 mm for 2D cultures and 5 mm for spheroid cultures. For each stimulation condition, 2D chondrocytes were cultured in 200 μl of complete medium per well, whereas cartilage spheroids were maintained in 200 μl of complete medium per well during stimulation. In the control (Ctrl) group, electrodes were placed in the same configuration but no current was applied, serving as a sham-stimulation control. Local electric-field distribution and current density were not directly mapped; therefore, potential spatial variations in electrical exposure cannot be completely excluded.

For all experiments, continuous direct microcurrent stimulation was applied for 30 min once daily. Cells or spheroids were assigned to 6 groups according to the applied current intensity: 0 μA (Ctrl), 10 μA, 25 μA, 50 μA, 100 μA, and 200 μA. The selected stimulation intensities were designed to establish a broad dose–response profile. Specifically, 10 and 25 μA represented low-intensity stimulation conditions, and 50 μA represented an intermediate stimulation level for identifying potentially beneficial biological effects, whereas 100 and 200 μA represented higher-intensity conditions to evaluate whether excessive electrical stimulation could attenuate regenerative responses or induce cellular stress. The polarity of the electrodes was kept consistent throughout the stimulation period. During stimulation, cultures were maintained under sterile conditions, and the medium was replaced every 2 to 3 d. To minimize potential nonspecific effects caused by electrochemical reactions and evaluate stimulation safety, temperature, pH, and ROS levels in the culture medium were monitored after prolonged stimulation as described below.

For 2D experiments, microcurrent stimulation was initiated after chondrocytes had attached overnight. For 3D experiments, stimulation was initiated 24 h after spheroid formation and continued for the indicated culture period. For mechanistic experiments, cartilage spheroids were further divided into the following groups: Ctrl (0 μA), 50 μA, 50 μA + verapamil, 50 μA + BAPTA-AM, 50 μA + CsA, and 200 μA. Verapamil (10 μM), BAPTA-AM (10 μM), or CsA (1 μM), all purchased from Sigma-Aldrich (USA), was added 30 min before microcurrent stimulation and maintained throughout the stimulation period.

### Two-dimensional culture model

For 2D experiments, auricular chondrocytes were seeded into 96-well plates at a density of 1 × 10^5^ cells/well and allowed to attach overnight before the initiation of microcurrent stimulation. The 2D model was used for DNA quantification, CCK-8 assay, Live/Dead staining, EdU assay, Ki67 immunofluorescence staining, RT-qPCR, Western blotting, and immunofluorescence staining of chondrogenic markers. According to the figure design, DNA content, EdU, Ki67, RT-qPCR, Western blotting, and immunofluorescence were analyzed at day 9, whereas the CCK-8 assay was measured at day 5 and from day 0 to day 11.

### DNA quantification

At day 9, total DNA content was measured using the Quant-iT PicoGreen dsDNA Assay Kit (Invitrogen, Thermo Fisher Scientific, USA) according to the manufacturer’s instructions. Cell lysates were mixed with PicoGreen working solution and incubated for 5 min at room temperature in the dark. Fluorescence was measured using a multimode microplate reader (Synergy H1, BioTek, Winooski, VT, USA) at emission wavelengths of 520 nm. DNA content was normalized to the 0-μA control group.

### CCK-8 assay

Cell metabolic activity was measured using the CCK-8 kit (Beyotime Biotechnology, Shanghai, China). Chondrocytes were seeded into 96-well plates at 5 × 10^5^ cells/well for the CCK-8 assay. At the indicated time points, the culture medium was replaced with fresh medium containing 10% CCK-8 reagent, and cells were incubated for 1 h at 37 °C. Absorbance (optical density) was measured at 450 nm using a microplate reader.

### Live/Dead staining

For 2D experiments, Live/Dead staining was performed at days 1 to 9. For 3D cartilage spheroids, Live/Dead staining was performed at days 3 and 7. Samples were washed with PBS and incubated in working solution containing 2 μM calcein-AM and 4 μM propidium iodide using a calcein-AM/propidium iodide Live/Dead staining kit (Beyotime Biotechnology, Shanghai, China) for 20 min at 37 °C in the dark. Fluorescence images were captured using an inverted fluorescence microscope (IX73, Olympus, Tokyo, Japan). Cell viability was quantified using ImageJ.

### EdU assay

At day 9, proliferative activity in the 2D culture model was assessed using the EdU Cell Proliferation Kit (Beyotime Biotechnology, Shanghai, China). Cells were incubated with 10 μM EdU for 2 h, fixed with 4% paraformaldehyde for 15 min, permeabilized with 0.3% Triton X-100 for 10 min, and reacted with the Click-iT staining solution according to the manufacturer’s protocol. Nuclei were counterstained with 4′,6-diamidino-2-phenylindole (DAPI; Sigma-Aldrich, USA). Images were acquired using a fluorescence microscope, and the percentage of EdU-positive cells was calculated.

### Ki67 immunofluorescence staining

At day 9, chondrocytes were fixed with 4% paraformaldehyde, permeabilized with 0.1% Triton X-100 for 10 min, and blocked with 5% bovine serum albumin (BSA) for 1 h. Samples were incubated overnight at 4 °C with anti-Ki67 primary antibody (ab15580, Abcam, Cambridge, UK, 1:200), followed by fluorescence-conjugated secondary antibody (Alexa Fluor 488 goat anti-rabbit IgG, Invitrogen, Carlsbad, CA, USA, 1:500) for 1 h at room temperature. Nuclei were stained with DAPI. Images were obtained using a confocal laser scanning microscope (FV3000, Olympus, Tokyo, Japan), and the Ki67-positive rate was quantified.

### RT-qPCR analysis

Total RNA was extracted using TRIzol reagent (Takara Bio, Shiga, Japan) according to the manufacturer’s protocol. RNA concentration and purity were determined using a NanoDrop spectrophotometer (Thermo Fisher Scientific, Waltham, MA, USA). Complementary DNA was synthesized from 1 μg of RNA using a reverse transcription kit. Quantitative PCR was performed using the SYBR Green Master Mix on the QuantStudio 5 Real-Time PCR System (Thermo Fisher Scientific, Waltham, MA, USA).

For 2D experiments, the relative mRNA expression levels of *Col2a1*, *SOX9*, and *ACAN* were measured at day 9. For the supplementary proliferation analysis in 3D spheroids, *Mki67*, *Pcna*, and *Ccnd1* were analyzed at days 3, 7, and 14. Relative expression levels were normalized to *glyceraldehyde-3-phosphate dehydrogenase* (*GAPDH*) and calculated using the 2^−ΔΔCt^ method. Primer sequences are listed in Table S1. All reactions were performed in triplicate.

### Western blot analysis

Total protein was extracted from chondrocytes or cartilage spheroids using radioimmunoprecipitation assay lysis buffer supplemented with protease and phosphatase inhibitor cocktail (Beyotime Biotechnology, Shanghai, China) according to the manufacturer’s instructions. Protein concentration was determined using a bicinchoninic acid protein assay kit (Beyotime Biotechnology, Shanghai, China). Equal amounts of protein (20 μg per sample) were mixed with 5× loading buffer, denatured at 95 °C for 5 min, and separated by 10% sodium dodecyl sulfate–polyacrylamide gel electrophoresis. The separated proteins were then transferred onto polyvinylidene fluoride membranes (Millipore, Billerica, MA, USA) using a wet transfer system (Trans-Blot Turbo Transfer System, Bio-Rad, Hercules, CA, USA). After transfer, membranes were blocked with 5% BSA in tris-buffered saline with Tween 20 (TBST) for 1 h at room temperature and then incubated overnight at 4 °C with the corresponding primary antibodies against COL II, SOX9, ACAN, cleaved caspase-3, PCNA, cyclin D1, NFATc1, Bax, Bcl-2, and β-actin. The antibodies were diluted according to the manufacturers’ recommendations, and a dilution ratio of 1:1,000 was used for most primary antibodies unless otherwise specified. β-Actin was used as the loading control.

After washing 3 times with TBST, membranes were incubated with the appropriate horseradish peroxidase-conjugated secondary antibodies (Cell Signaling Technology, Danvers, MA, USA, 1:5,000) for 1 h at room temperature. Protein bands were visualized using an enhanced chemiluminescence detection kit (Thermo Fisher Scientific, Waltham, MA, USA) and captured using a chemiluminescence imaging system (ChemiDoc MP, Bio-Rad, Hercules, CA, USA). Band intensities were quantified using the ImageJ software and normalized to β-actin.

### Immunofluorescence staining

Immunofluorescence staining was performed to evaluate the expression of chondrogenic, dedifferentiation-related, catabolic, inflammatory, and signaling-related proteins in chondrocytes and cartilage spheroids. For 2D cultured chondrocytes, immunofluorescence staining of COL II, COMP, and ACAN was performed at day 9. For 3D cartilage spheroids, immunofluorescence staining of COL II, COMP, ACAN, COL I, COL X, RUNX2, MMP13, ADAMTS5, iNOS, and COX-2 was performed at day 12. In addition, NFATc1 immunofluorescence staining was performed in cartilage spheroids from the 0 μA, 50 μA, 50 μA + verapamil, 50 μA + BAPTA-AM, 50 μA + CsA, and 200 μA groups at day 12 to evaluate NFAT nuclear translocation.

Briefly, samples were fixed with 4% paraformaldehyde for 20 min at room temperature, washed 3 times with PBS, and permeabilized with 0.3% Triton X-100 for 15 min. After washing, the samples were blocked with 5% BSA for 1 h at room temperature. The samples were then incubated overnight at 4 °C with the corresponding primary antibodies against COL II, COMP, ACAN, COL I, COL X, RUNX2, MMP13, ADAMTS5, iNOS, COX-2, or NFATc1. Unless otherwise specified, primary antibodies were diluted at 1:200 in blocking buffer. After 3 washes with PBS, samples were incubated with the appropriate fluorescence-conjugated secondary antibodies (1:500) for 1 h at room temperature in the dark. Nuclei were counterstained with DAPI for 5 min.

For 2D chondrocytes, fluorescence images were acquired using an inverted fluorescence microscope. For cartilage spheroids, paraffin with a thickness of 6 μm was prepared before staining, and images were acquired using a confocal laser scanning microscope under identical acquisition settings for all groups.

Quantitative analysis was performed using the ImageJ software. For COL II, COMP, ACAN, COL I, COL X, RUNX2, MMP13, ADAMTS5, iNOS, and COX-2, the fluorescence-positive area was measured and normalized to the corresponding field area. For NFATc1, the nuclear/cytoplasmic fluorescence ratio was calculated based on DAPI-defined nuclear regions, and the percentage of cells with nuclear NFAT localization was further quantified.

### Formation and culture of engineered cartilage spheroids

Engineered cartilage spheroids were established through spontaneous self-assembly of chondrocytes to evaluate the effects of microcurrent stimulation in a 3D culture system. Briefly, auricular chondrocytes were harvested and resuspended in complete culture medium at an appropriate density. An equal number of cells (1 × 10^5^ cells per spheroid) was seeded into nonadhesive spheroid molds to ensure consistent spheroid formation among experimental groups. Cells were allowed to self-aggregate for 24 h under standard culture conditions (37 °C, 5% CO_2_) before the initiation of microcurrent stimulation. During the subsequent culture period, one spheroid was maintained per well.

For dose-dependent evaluation, cartilage spheroids were divided into 6 groups: 0 μA (Ctrl), 10 μA, 25 μA, 50 μA, 100 μA, and 200 μA. Microcurrent stimulation was applied for 30 min once daily for a total culture period of 12 d. The culture medium was replaced every 2 to 3 d throughout the experiment. Spheroid morphology was monitored by light microscopy, and representative images were recorded at the indicated time points. At day 12, spheroids were collected for morphological analysis, histological staining, immunohistochemistry, immunofluorescence staining, biochemical quantification of matrix components, mechanical testing, and protein analysis.

For mechanistic experiments involving Ca^2+^/calcineurin/NFAT signaling, cartilage spheroids were further assigned to the 0 μA, 50 μA, 50 μA + verapamil, 50 μA + BAPTA-AM, 50 μA + CsA, and 200 μA groups. Verapamil (10 μM), BAPTA-AM (10 μM), or CsA (1 μM) was added 30 min before microcurrent stimulation in the corresponding inhibitor groups. These spheroids were then used for Fluo-4 AM calcium imaging, NFAT immunofluorescence staining, and Western blot analysis of NFATc1.

To evaluate in vivo cartilage-forming potential, spheroids generated under the 0-, 50-, and 200-μA conditions were collected after in vitro stimulation, resuspended in sterile PBS, and used for subsequent subcutaneous injection into nude mice.

### Histology and immunohistochemistry

For histological and immunohistochemical evaluation, cartilage spheroids cultured in vitro for 12 d and explanted samples retrieved after 2 weeks of subcutaneous implantation were collected, washed with PBS, and fixed in 4% paraformaldehyde for 24 h at 4 °C. After fixation, samples were dehydrated through a graded ethanol series, cleared in xylene, embedded in paraffin, and sectioned at a thickness of 6 μm using a rotary microtome (RM2235, Leica Biosystems, Wetzlar, Germany). The sections were mounted on glass slides and dried at 42 °C before staining.

For H&E staining, paraffin sections were deparaffinized in xylene, rehydrated through graded ethanol, and stained with hematoxylin for 3 min and eosin for 1 min according to standard protocols. For TB staining, rehydrated sections were stained with TB solution (Solarbio, Beijing, China) for 2 min to visualize sulfated GAGs. For Safranin O staining, sections were stained using a Safranin O/Fast Green kit (Servicebio, Wuhan, China) according to the manufacturer’s instructions to evaluate proteoglycan-rich cartilage matrix. Stained sections were rinsed, dehydrated, cleared, and mounted with neutral resin. Representative enlarged views of H&E and Safranin O staining were further obtained for detailed morphological comparison.

For COL II immunohistochemistry, deparaffinized and rehydrated sections were subjected to antigen retrieval in citrate buffer (10 mM, pH 6.0) using a water bath (95 °C for 15 min), followed by cooling to room temperature. Endogenous peroxidase activity was blocked with 3% hydrogen peroxide for 10 min, and nonspecific binding was blocked with 5% BSA for 30 min at room temperature. Sections were then incubated overnight at 4 °C with anti-COL II primary antibody (Abcam, ab34712, 1:200). After washing with PBS, sections were incubated with the appropriate horseradish peroxidase-conjugated secondary antibody (Cell Signaling Technology, 1:500) for 30 min at room temperature. Immunoreactivity was visualized using 3,3′-diaminobenzidine substrate (Vector Laboratories, Burlingame, CA, USA), and nuclei were counterstained with hematoxylin. All stained sections were imaged using a bright-field microscope (BX53, Olympus, Tokyo, Japan) equipped with a digital camera. For quantitative analysis, the positive staining area or staining intensity of COL II and the histological appearance of TB and Safranin O staining were analyzed using the ImageJ software under identical imaging settings. Histological images and quantitative analyses were performed by investigators blinded to the treatment groups. For in vivo samples, the right panels shown in the corresponding figure represent enlarged views of the selected regions from the left panels.

### Quantification of GAG, COL II, and DNA

Total GAG content was measured using a dimethylmethylene blue assay kit (Biocolor Ltd., Carrickfergus, UK). According to the manufacturer’s instructions. Total COL II content was determined using an enzyme-linked immunosorbent assay kit (Solarbio, Beijing, China). Total DNA content was measured using the PicoGreen dsDNA assay. For normalized analysis, COL II/DNA and GAG/DNA ratios were calculated.

### ATP-based metabolic activity in cartilage spheroids

The ATP-based metabolic activity of cartilage spheroids was assessed at days 3, 7, and 14 using the CellTiter-Glo 3D Cell Viability Assay (Promega, Madison, WI, USA). Individual spheroids were transferred into white 96-well plates, and an equal volume of CellTiter-Glo 3D reagent was added to each well. After incubation on an orbital shaker for 5 min and stabilization at room temperature for 20 min, luminescence was measured using a multimode plate reader. Results were normalized to the control group.

### TUNEL staining

Apoptosis in cartilage spheroids was evaluated at days 3 and 7 using a TUNEL apoptosis detection kit (Roche Diagnostics, Basel, Switzerland) according to the manufacturer’s instructions. TUNEL-positive nuclei were visualized using a fluorescence microscope and quantified as a percentage of total DAPI-positive nuclei.

### Bax/Bcl-2 and cleaved caspase-3 analysis

At day 12, apoptosis-related protein expression in cartilage spheroids was evaluated by Western blotting for cleaved caspase-3, Bax, and Bcl-2, and the Bax/Bcl-2 ratio was calculated.

### Assessment of microenvironmental changes associated with electrochemical side reactions

To evaluate possible electrochemical side effects of prolonged microcurrent stimulation, the temperature, culture medium pH, and ROS levels of the system were measured after 9 d of stimulation. Temperature was measured using a digital thermometer (HI98509, Hanna Instruments, Woonsocket, RI, USA), and pH was measured using a micro-pH meter (S20 SevenCompact, Mettler-Toledo, Greifensee, Switzerland). ROS levels were measured using a ROS detection kit (Beyotime Biotechnology, Shanghai, China) according to the manufacturer’s instructions.

### Mechanical characterization of cartilage spheroids

At day 12, the compressive modulus and dynamic storage modulus of cartilage spheroids were measured using a mechanical testing system (Instron, Norwood, MA, USA). For compressive testing, spheroids were compressed at a displacement rate of 0.5 mm/min, and the compressive modulus was calculated from the linear region of the stress–strain curve. For dynamic testing, oscillatory loading was applied at 1 Hz with a strain amplitude of 5%, and the storage modulus was calculated.

For water content and wet-to-dry ratio, spheroids were weighed immediately after gentle blotting to obtain wet weight and then freeze-dried for 24 h to obtain dry weight. Water content was calculated as (wet weight − dry weight)/wet weight × 100%, and the wet-to-dry weight ratio was calculated as wet weight/dry weight.

### Fluo-4 AM calcium imaging

Intracellular Ca^2+^ dynamics were evaluated in cartilage spheroids from the 0-, 50-, and 200-μA groups using Fluo-4 AM (Invitrogen, Thermo Fisher Scientific, Waltham, MA, USA). For inhibitor experiments, cartilage spheroids were further assigned to the 50 μA + verapamil, 50 μA + BAPTA-AM, and 50 μA + CsA groups. Verapamil (10 μM), BAPTA-AM (10 μM), or CsA (1 μM) was added 30 min before microcurrent stimulation in the corresponding inhibitor groups.

Briefly, cartilage spheroids were incubated with 5 μM Fluo-4 AM in serum-free medium containing 0.02% Pluronic F-127 at 37 °C for 30 min in the dark. After dye loading, spheroids were washed 3 times with PBS or phenol red-free medium and further incubated in dye-free medium for 20 min at 37 °C to allow complete de-esterification. Time-lapse calcium imaging was then performed using a confocal laser scanning microscope equipped with a live-cell imaging chamber (LSM 880 with Airyscan, Zeiss, Oberkochen, Germany) at 488-nm excitation. Images were acquired at an interval of 0.8 s/frame during microcurrent stimulation.

Fluorescence intensity was analyzed as Δ*F*/*F*_0_, where *F*_0_ represents the baseline fluorescence intensity before stimulation and *F* represents the fluorescence intensity at each time point after stimulation. Representative imaging snapshots were selected at the respective peak-response time points. The Ca^2+^ signal AUC was calculated from the Δ*F*/*F*_0_–time curve. A responsive cell was defined as a cell showing a fluorescence increase of Δ*F*/*F*_0_ > 10% within 60 s after stimulation, and the responsive cell ratio was calculated as the percentage of responsive cells among the total analyzed cells. Quantitative analysis was performed using ImageJ and GraphPad Prism software.

### NFAT nuclear translocation assay

NFAT nuclear translocation was analyzed in cartilage spheroids from the 0 μA, 50 μA, 50 μA + verapamil, 50 μA + BAPTA-AM, 50 μA + CsA, and 200 μA groups at day 12. Verapamil (10 μM), BAPTA-AM (10 μM), or CsA (1 μM) was added 30 min before microcurrent stimulation in the corresponding inhibitor groups.

For immunofluorescence staining, cartilage spheroids were fixed in 4% paraformaldehyde for 20 min, permeabilized with 0.3% Triton X-100 for 15 min, and blocked with 5% BSA for 1 h at room temperature. Samples were then incubated overnight at 4 °C with an anti-NFATc1 primary antibody (sc-7294, Santa Cruz Biotechnology, Dallas, TX, USA, 1:200), followed by incubation with the corresponding fluorescence-conjugated secondary antibody (Thermo Fisher Scientific, 1:500) for 1 h at room temperature in the dark. Nuclei were counterstained with DAPI for 5 min. Fluorescence images were acquired using a confocal laser scanning microscope under identical acquisition settings for all groups.

NFAT nuclear translocation was quantified by calculating the nuclear/cytoplasmic fluorescence ratio using ImageJ. Briefly, the nuclear region was identified based on DAPI staining, and the mean fluorescence intensity of NFATc1 in the nucleus and cytoplasm was measured for each cell. In addition, the percentage of cells with nuclear NFAT localization was quantified as the proportion of cells showing predominant NFATc1 fluorescence within the nuclear region. For biochemical validation, total NFATc1 protein expression in cartilage spheroids at day 12 was further evaluated by Western blotting as described above.

### Subcutaneous implantation of cartilage spheroid suspensions

To assess the in vivo cartilage-forming potential of microcurrent-treated spheroids, cartilage spheroids from the 0-, 50-, and 200-μA groups were collected after in vitro stimulation and gently resuspended in sterile PBS or saline. For each implantation site, 20 spheroids, corresponding to approximately 2 × 10^6^ total cells, were suspended in 200 μl of sterile PBS or saline to ensure that the same number of spheroids was implanted in each animal.

The spheroid suspension was injected subcutaneously into the dorsal region of male BALB/c nude mice aged 4 weeks (Shanghai Yunde Biotechnology Co., Ltd., China) using a sterile 1-ml syringe fitted with a 22 G needle. Each mouse received one subcutaneous injection site, with an injection volume of 200 μl per site. Animals were randomly assigned to different treatment groups, with 5 animals used for each group (*n* = 5). After 2 weeks of implantation, the samples were harvested for gross observation, histological staining, COL II immunohistochemistry, biochemical quantification, and mechanical testing.

### Statistical analysis

All quantitative data are presented as mean ± standard deviation. Biological replicates were used for statistical analysis, and technical replicates were averaged before analysis. No data were excluded unless caused by technical failure. Statistical analyses were performed using GraphPad Prism. Comparisons among multiple groups were performed using one-way analysis of variance (ANOVA) followed by Tukey’s post hoc test. For experiments involving both group and time as independent variables, 2-way ANOVA followed by Tukey’s post hoc test was used. A value of *P* < 0.05 was considered statistically significant.

## Ethical Approval

All animal procedures were approved by the Institutional Animal Care and Use Committee of Shanghai Pulmonary Hospital (approval no. K21-355Y) and were conducted in accordance with institutional guidelines.

## Data Availability

The datasets generated and/or analyzed during the current study are available from the corresponding authors upon reasonable request.
