# Supplementary material for: Effect of Microcurrent Stimulation on Cartilage Regeneration in Engineered Chondrocyte Spheroids
Source: Research (Wash D C). 2026 Oct 19;9:1462. doi: 10.34133/research.1462 (PMC13678085; doi:10.34133/research.1462)
Supplement: Supplementary file 1 [file research.1462.f1.docx]

**Supplemental Information**

**Effect of Microcurrent Stimulation on Cartilage Regeneration in Engineered Chondrocyte Spheroids**

*Yong Xu^#^, Siqiang Zheng^#^, Qiangsheng Hu^*^, Bo Tao^*^*

Department of Thoracic Surgery, Shanghai Pulmonary Hospital, School of Medicine, Tongji University, Shanghai, China.

^#^ These authors contributed equally to this work.

* Corresponding authors: Dr. Qiangsheng Hu, Email: 2011085@tongji.edu.cn; Dr. Bo Tao, Email: tbo0820@163.com.

**Figure S1. *In vitro* assessment of chondrocyte metabolic/viability activity under microcurrent stimulation.** Relative optical density (OD) values of chondrocytes in the control (Ctrl, 0 μA), 10, 25, 50, 100, and 200 μA groups from day 0 to day 11, as measured by the CCK-8 assay. **Data are presented as mean ± SD (n = 5).**

**Figure S2. Assessment of possible microenvironmental changes associated with electrochemical side reactions following microcurrent stimulation.** **(A)** Temperature levels in the control (Ctrl, 0 μA), 10, 25, 50, 100, and 200 μA groups at 1, 5, and 9 days of microcurrent stimulation. **(B)** Culture medium pH values in the Ctrl (0 μA), 10, 25, 50, 100, and 200 μA groups at 1, 5, and after 9 days of microcurrent stimulation. **(C)** Reactive oxygen species (ROS) levels in the Ctrl, 10, 25, 50, 100, and 200 μA groups after 9 days of microcurrent stimulation. **Data are presented as mean ± SD (n = 5). ns, not significant.**

**Figure S3. Quantitative analysis of the volume shrinkage rate of cartilage spheroids in the control (Ctrl, 0 μA), 10, 25, 50, 100, and 200 μA groups at day 12. Data are presented as mean ± SD (n = 5). ns, not significant; **P* < 0.05, ***P* < 0.01.**

**Figure S4. Physicochemical and mechanical characterization of microcurrent-treated cartilage spheroids. Quantitative analysis of cartilage spheroid properties, including (A) compressive modulus, (B) dynamic storage modulus, (C) water content, and (D) wet-to-dry weight ratio of cartilage spheroids in the control (Ctrl, 0 μA), 10, 25, 50, 100, and 200 μA groups at day 12.** **Data are presented as mean ± SD (n = 5). ns, not significant; ***P **< 0.05, ****P **< 0.01, ******P **< 0.0001.**

**Figure S5.** Representative enlarged images of H&E and Safranin O staining of cartilage spheroids in the control (Ctrl, 0 μA), 10, 25, 50, 100, and 200 μA groups at day 12.

**Figure S6. Relative mRNA expression levels of proliferation-related genes, including *Mki67*, *Pcna*, and *Ccnd1*, in the control (Ctrl, 0 μA), 10, 25, 50, 100, and 200 μA groups at day 3, 7, and 14, as determined by RT-qPCR.** **Data are presented as mean ± SD (n = 5). ns, not significant.**

**Figure S7. Representative Live/Dead staining images of cartilage spheroids in the control (Ctrl, 0 μA), 10, 25, 50, 100, and 200 μA groups at days 3 and 7.**

**Figure S8. TUNEL staining analysis of apoptosis in cartilage spheroids. (A-B) Representative TUNEL staining images of cartilage spheroids in the control (Ctrl, 0 μA), 10, 25, 50, 100, and 200 μA groups at days 3 and 7. (C-D) Corresponding quantitative analysis of TUNEL-positive cells in each group at days 3 and 7. Data are presented as mean ± SD (n = 5). ns, not significant.**

**Figure S9. Bax and Bcl-2 expression in cartilage spheroids following microcurrent stimulation. (A)** Representative Western blot images showing the protein expression levels of Bax and Bcl-2 in cartilage spheroids from the control (Ctrl, 0 μA), 10, 25, 50, 100, and 200 μA groups at day 12, with β-actin used as the loading control. Corresponding quantitative analysis of the relative protein expression levels of **(B)** Bax, **(C)** Bcl-2, and **(D)** the Bax/Bcl-2 ratio in each group at day 12. Data are presented as mean ± SD (n = 5). ns, not significant; *P < 0.05, ****P < 0.0001.

**Figure S10.** Quantitative analysis of the percentage of cells with nuclear NFAT localization in cartilage spheroids from the 0 μA, 50 μA, 50 μA + Verapamil, 50 μA + BAPTA-AM, 50 μA + CsA, and 200 μA groups at day 12. Data are presented as mean ± SD (n = 5). ns, not significant; **P* < 0.05, *****P* < 0.0001.

**Table S1. Primer sequences used for RT-qPCR**

| **Genes** | **Forward primer (5′-3′)** | **Reverse primer (5′-3′)** |
| --- | --- | --- |
| *Col2a1* | ATCCAGGGCTCCAATGA | TCTTCTGGAGTGCGGAA |
| *SOX9* | CCAGAGAACGCACATCAAG | ATACTGATGTGGCTGGTGG |
| *ACAN* | CAAGTGGAGCCGTGTTT | TTTAGGTCTTGGAAGCGAG |
| *Mki67* | CCACACTGTGTCGTCGTTTG | CCGTGCGCTTATCCATTCA |
| *Pcna* | ACAGAGCATGGATTCGTCTCAC | AGAAAACTTCACCCCGTCCTTT |
| *Ccnd1* | GGAGCAGAAGTGCGAAGA | GGGTGGGTTGGAAATGAA |
| *GAPDH* | TGCCAAATATGATGACATCAAGAA | GGAGTGGGTGTCGCTGTTG |
